# Genome-Wide Identification of Peanut *KCS* Genes Reveals That *AhKCS1* and *AhKCS28* Are Involved in Regulating VLCFA Contents in Seeds

**DOI:** 10.3389/fpls.2020.00406

**Published:** 2020-05-07

**Authors:** Dongxin Huai, Xiaomeng Xue, Yang Li, Peng Wang, Jianguo Li, Liying Yan, Yuning Chen, Xin Wang, Nian Liu, Yanping Kang, Zhihui Wang, Yi Huang, Huifang Jiang, Yong Lei, Boshou Liao

**Affiliations:** ^1^Key Laboratory of Biology and Genetic Improvement of Oil Crops, Ministry of Agriculture and Rural Affairs, Oil Crops Research Institute of Chinese Academy of Agricultural Sciences, Wuhan, China; ^2^College of Life Sciences, Henan Agricultural University, Zhengzhou, China; ^3^Key Laboratory of Crop Gene Resources and Germplasm Enhancement in Southern China, Ministry of Agriculture and Rural Affairs, Danzhou, China; ^4^Tropical Crops Genetic Resources Institute, Chinese Academy of Tropical Agricultural Sciences, Danzhou, China

**Keywords:** peanut, β-ketoacyl-CoA synthase (KCS), very long chain fatty acid (VLCFA) biosynthesis, expression profiling, function analysis

## Abstract

The peanut (*Arachis hypogaea* L.) is an important oilseed crop worldwide. Compared to other common edible vegetable oils, peanut oil contains a higher content of saturated fatty acids (SFAs), approximately 20–40% of which are very long chain fatty acids (VLCFAs). To understand the basis for this oil profile, we interrogated genes for peanut β-ketoacyl-CoA synthase (KCS), which is known to be a key enzyme in VLCFA biosynthesis. A total of 30 *AhKCS* genes were identified in the assembled genome of the peanut. Based on transcriptome data, nine *AhKCS* genes with high expression levels in developing seeds were cloned and expressed in yeast. All these AhKCSs could produce VLCFAs but result in different profiles, indicating that the AhKCSs catalyzed fatty acid elongation with different substrate specificities. Expression level analysis of these nine *AhKCS* genes was performed in developing seeds from six peanut germplasm lines with different VLCFA contents. Among these genes, the expression levels of *AhKCS1* or *AhKCS28* were, 4–10-fold higher than that of any other *AhKCS*. However, only the expression levels of *AhKCS1* and *AhKCS28* were significantly and positively correlated with the VLCFA content, suggesting that AhKCS1 and AhKCS28 were involved in the regulation of VLCFA content in the peanut seed. Further subcellular localization analysis indicated that AhKCS1 and AhKCS28 were located at the endoplasmic reticulum (ER). Overexpression of *AhKCS1* or *AhKCS28* in *Arabidopsis* increased the contents of VLCFAs in the seed, especially for very long chain saturated fatty acids (VLCSFAs). Taken together, this study suggests that *AhKCS1* and *AhKCS28* could be key genes in regulating VLCFA biosynthesis in the seed, which could be applied to improve the health-promoting and nutritional qualities of the peanut.

## Introduction

The peanut (*Arachis hypogaea* L.) is a widely cultivated oilseed crop in tropical and subtropical regions, which provides a significant source of protein, folate, tocopherol, phytosterols, polyphenolics such as resveratrol, fiber, and edible oil (Bertioli et al., 2015; Han, 2016; Bertioli et al., 2019). Fatty acid composition is the main factor in determining the nutritional value and application purpose of vegetable oil (Napier and Graham, 2010; Napier et al., 2014). Peanut oil contains ∼20% saturated fatty acids (SFAs), which is higher than that in many commercial vegetable oils (Wang et al., 2013; Giakoumis, 2018). Limiting the intake of dietary SFAs is recommended by most dietary guidelines, because SFAs promote increasing undesirable low-density lipoprotein (LDL) cholesterol in the blood, leading to a high risk of cardiovascular disease (List, 2004; USDA, 2010; Briggs et al., 2017). In the peanut, SFAs are enriched in VLCSFAs, including arachidic acid (C20:0), behenic acid (C22:0), and lignoceric acid (C24:0) (Wang et al., 2013; Giakoumis, 2018). VLCFAs are fatty acids longer than 18 atoms (Haslam and Kunst, 2013; Giakoumis, 2018). Hence, reducing VLCFA content in the peanut is a prime target to improve the nutritional value of peanuts and peanut oil.

In plants, it has been proved that VLCFA biosynthesis is controlled by a condensing enzyme: β-ketoacyl-CoA synthase (KCS) (Todd et al., 1999; Sassa and Kihara, 2014; Huai et al., 2015; Usher et al., 2017). KCS catalyzes the first step of elongation with the substrate and tissue specificities (Blacklock and Jaworski, 2002, 2006; Haslam and Kunst, 2013; Huai et al., 2015). The expression level and substrate preference of KCS determine the ultimate chain length and contents of VLCFAs (James et al., 1995; Katavic et al., 2001; Denic and Weissman, 2007; Sun et al., 2013; Huai et al., 2015; Shi et al., 2017; Ozseyhan et al., 2018). For example, the *KCS* from *Lunaria annua* has been introduced into camelina (*Camelina sativa*) to produce nervonic acid (C24:1) (Guo et al., 2009; Huai et al., 2015). The *fatty acid elongation 1* (*FAE1*) from *Brassica napus* is overexpressed in the rapeseed to increase the content of erucic acid (C22:1) (Nath et al., 2009; Li et al., 2012). In contrast, the silencing of *FAE1* is used to decrease the contents of VLCFAs (Wang et al., 2010; Shi et al., 2017; Ozseyhan et al., 2018).

According to sequence similarity, *KCS* genes in higher plants are divided into two gene families, namely FAE1-type and elongation-type (ELO-type) (Haslam and Kunst, 2013; Guo et al., 2016). A total of 21 FAE1-type KCSs were identified in the *Arabidopsis* genome (Joubes et al., 2008). As the expression patterns and substrate specificities of KCSs are different, they are assigned to play different roles in seed oil biosynthesis (FAE1/KCS18) (James and Dooner, 1990; Lemieux et al., 1990; Kunst et al., 1992; James et al., 1995), development of epidermis (FDH/KCS10, HIC/KCS13) (Gray et al., 2000; Pruitt et al., 2000), suberin metabolism (KCS2/DAISY, KCS9, KCS20) (Franke et al., 2009; Lee et al., 2009; Kim J. et al., 2013), and cuticular lipid metabolism (CER6/CUT1/KCS6, KCS1, KCS16) (Todd et al., 1999; Fiebig et al., 2000; Hegebarth et al., 2017). The ELO-type family is homologous to animal condensing enzymes but shares little homology with the plant KCS enzymes (Leonard et al., 2004; Haslam and Kunst, 2013). Four ELO genes are characterized in *Arabidopsis* (Quist et al., 2009). Only HOS3 is reported to be involved in the biosynthesis of sphingolipid (Quist et al., 2009), while the functions of other plant ELO homologs remain to be investigated (Haslam and Kunst, 2013).

To reduce the contents of VLCFAs in the peanut, it is necessary to identify which *KCS* genes contribute to this trait. In this report, we identified *AhKCS* genes in the peanut, and investigated the expression profiles of these genes. The coding sequences (CDS) and expression levels of *AhKCS* genes in developing seeds were analyzed in peanut lines with different VLCFA contents. Correlation analysis between the *AhKCS* gene expression and VLCFA content identified the major genes contributing to VLCFA content. The identified genes were heterologously expressed in yeast and *Arabidopsis* to confirm their function and substrate specificities. Finally, the candidate AhKCSs for the seed VLCFA content were identified which could be applied in the improvement of peanut oil.

## Materials and Methods

### Plant Materials and Growth Conditions

Peanut (*Arachis hypogaea* L.) cultivar Zhonghua16, germplasm lines C-34, C-119, C-140, C-178, C-224, and C-296 were maintained in our lab. Peanut plants were sowed in the Oil Crops Research Institute of the Chinese Academy of Agricultural Sciences (OCRI-CAAS) experimental field in Wuhan, China. Plants were grown under field conditions in a randomized block experimental design with three replications.

*Arabidopsis* (*Arabidopsis thaliana*) double mutant *fae1/fad2* (Smith et al., 2003) was obtained from Dr. Edgar Cahoon’s Lab at the University of Nebraska-Lincoln, United States. *Arabidopsis* plants were grown in controlled-environment chambers with 16 h light (21°), 8 h dark (18°) cycle, 100 mE light intensity, and 60% relative humidity. Double mutant *fae1/fad2* as control plants were included in each flat area to minimize any spatial aspects of the growth chamber.

### Identification of *AhKCS* Genes

Datasets of the genome sequence were downloaded from the following sources. *Arachis hypogaea*: PeanutBase^1^ (Bertioli et al., 2019); *Arabidopsis thaliana*: TAIR^2^ (Lamesch et al., 2011).

*Arabidopsis KCS* genes were downloaded in TAIR, whose Pfam domains were retrieved from Pfam Database^3^. Standalone similarity searches for peptide sequences were performed through BLASTP under BLAST + executable suite (Camacho et al., 2009). In addition, an HMM search was performed with the “trusted cutoff” as the threshold for detecting the domains. The results of the two rounds of searches were merged, which was subject to another search against a library of Pfam-A families.

### Genomic Distribution of AhKCSs

The chromosomal location and syntenic gene pairs information of *KCS* genes were downloaded from PeanutBase. The syntenic diagram illustrating the detailed genomic distribution of *KCS* genes was created by Circos^4^ (Krzywinski et al., 2009).

### Alignment and Phylogeny Inference

Multiple sequence alignments were performed using the Clustal W program (Thompson et al., 1994). A phylogenetic tree was constructed using the neighbor-joining (NJ) method in the MEGA X software with 1,000 bootstrap replicates (Kumar et al., 2018).

### Transcriptomic Analysis

Raw transcriptome data of 22 different tissues collected from cultivated peanut cv. Tifrunner were downloaded from NCBI (BioSample IDs: SAMN03944933-SAMN03944990) (Clevenger et al., 2016). Reads were mapped to the assembled genome of *A. hypogaea* which were deposited in-house. Read mapping and calculation of fragments per kilobase per million reads mapped (FPKM) were performed using TopHat2 (Kim D. et al., 2013). A heatmap was generated using pheatmap, an R package^5^.

### RNA Extraction

For investigation of the expression profile in developing seeds, seeds of Zhonghua 16 from five specific stages were collected without pericarp: I – white and flat embryo [approximately 20 days after pollination (DAP)]; II – white and teardrop-shaped embryo (30 DAP); III – white and torpedo to round shaped embryo (40 DAP); IV – light pink and round embryo (50 DAP); V – dark pink, large and round embryo (60 DAP). For association analysis between *KCS* expression level and VLCFA contents, developing seeds were harvested at stage III from germplasm lines C-34, C-119, C-140, C-178, C-224, and C-296. Total RNA was extracted using TRIzol reagent (Sigma)^6^ according to the manufacturer’s instructions. Reverse transcription was performed using SuperScript IV First-Strand Synthesis System as described by the manufacturer (Invitrogen)^7^.

### Quantitative Real-Time PCR (qRT-PCR) Analysis

Primers for qRT-PCR were designed using the IDT DNA Real Time PCR primer design tool^8^ (Supplementary Table S1). Real-time PCRs were performed using paired samples with three technical replicates on a Bio-Rad CFX96 Real-Time system (Bio-Rad)^9^ and DBI Bioscience Bestar-Real Time PCR Master Mix kit following the manufacturer’s instructions. The data were analyzed with LINREG as previously described (Huai et al., 2015). The experiment was repeated using at least three independent biological replicates, with three technical replicates for each biological sample.

### Expression of *AhKCS* Genes in Yeast

The nine *AhKCS* genes were individually amplified by PCR from sequenced TA-clones using the primers in Supplementary Table S2, with different restriction sites. The PCR products were digested with corresponding restriction enzymes to the added sites in primers, respectively. The digested fragments were ligated into the vector pYX242, respectively (Supplementary Figure S1). Based on the inserted gene, the resulting plasmids were designated as pYX242-AhKCS1, pYX242-AhKCS4, pYX242-AhKCS10, pYX242-AhKCS13, pYX242-AhKCS17, pYX242-AhKCS23, pYX242-AhKCS25, pYX242-AhyKCS28, and pYX242-AhKCS29.

The vectors with *AhKCS* genes were introduced into *Saccharomyces cerevisiae* strain INVSc1 (Invitrogen) using the S.c. EasyComp ^TM^ transformation Kit (Invitrogen). Yeast cells transformed with a pYX242 empty vector were used as the control. The transformed yeast cells were selected on minimal agar plates lacking leucine. Transformants were first grown in SC-Leu (synthetic complete minus leucine) medium at 28°C overnight, suspended in SC-Leu medium and grown at 28°C for 2 days; then the transformants were selected and grown as described previously (Guo et al., 2009).

Yeast cells were grown in 50 ml SC-Leu medium at 28°C overnight to OD_600_ of 1.4, the cells were spun to form a pellet and used for biochemical analysis. Cell pellets were lyophilized and then transferred into glass tubes. Fatty acid methyl esters (FAMEs) were prepared with 2.5% (v/v) sulfuric acid/methanol for gas-chromatography analysis as previously described (Huai et al., 2015).

### Cloning of *AhKCS* Genes From Peanut Plants

The *AhKCS* genes were cloned from developing seeds at stage III of peanut germplasm lines C-34, C-119, C-140, C-178, C-224, and C-296. Primers were designed based on the sequences of *Arahy.IFJ1V3*, *Arahy.T9PLK1*, *Arahy.1FMC3R*, *Arahy.WQ1I1V*, *Arahy.TIY3DH*, *Arahy.3ATP19, Arahy.XI5WK7*, *Arahy.BGR17W*, and *Arahy.YW30D2*. All the cloning primers are listed in Supplementary Table S3.

### Subcellular Localization of *AhKCS* Genes

The *AhKCS1* and *AhKCS28* genes without a stop codon were amplified by PCR from sequenced TA-clones using the following primers: 5′- CATGGGTACCATGGCTGATGCAAAAGCA-3′ (*Kpn*I) and 5′-CATGGGATCCTGATGGCAGATACCCTTGGA-3′ (*Bam*HI) (the added restriction sites are underlined). The PCR products were digested with *Kpn*I and *Bam*HI, and the digested fragments were separately ligated into the vector pHBT (GenBank accession No. EF090408). As a result, *AhKCS* genes were inserted between the cauliflower mosaic virus (CaMV) 35S promoter and the green fluorescent protein (GFP) gene. The resulting plasmids were designated as pHBT-AhKCS1-GFP and pHBT-AhKCS28-GFP. The empty vector pHBT-GFP was analyzed as a control. The *BnaA.FAE1* from rapeseed was fused with the red fluorescent protein (RFP) gene. The *p35S*:BnaA.FAE1-RFP construct was used as an endoplasmic reticulum (ER) marker (Wang et al., 2010; Haslam and Kunst, 2013).

The pHBT-GFP, pHBT-AhKCS1-GFP, and pHBT-AhKCS28-GFP were each transiently co-expressed with the ER marker in *Arabidopsis* protoplasts by PEG transformation, respectively (Nelson et al., 2007). The protoplast cells were isolated from 15-days-old seedlings of wild-type *Arabidopsis*. The leaf tissues were incubated in a solution containing 1.5% cellulase and 0.75% macerozyme for 4 h with gentle agitation. The protoplasts were collected by centrifuging at 100 g at 4°C for 2 min and resuspended in a solution of 0.4 M mannitol, 15 mM MgCl_2_, and 4 mM MES (pH 5.7). Then, 100 μl of the protoplast solution with 10 μg plasmid DNA was used for PEG-mediated transformation. After 10 h of incubation in the dark, fluorescence was examined under a laser-scanning confocal microscope (Olympus FV10-ASW).

### Expression of *AhKCS* Genes in Arabidopsis

The *AhKCS1* and *AhKCS28* genes were amplified by PCR from sequenced TA-clones using the following primers with added *Not*I restriction sites: 5′-CATGGCGGCCGCATGGCTGATGCAAAAGCA-3′ and 5′-CATGGCGGCCGCTCAGATGGCAGATACCCTTGGA-3′ (the added restriction sites are underlined). The *Not*I digested fragments were separately inserted into the vector pKMS2 (Huai et al., 2015) between the seed-specific soybean oleosin-1 promoter and 3′UTR, to create pKMS2-AhKCS1 and pKMS2-AhKCS28. The cassettes comprising the oleosin promoter and 3′UTR flanking AhKCS1 or AhKCS28 gene were excised using *Asc*I and respectively inserted into the binary vector pBinGlyRed2 containing a DsRed marker gene (Huai et al., 2015) to generate pBinGlyRed2-AhKCS1 and pBinGlyRed2-AhKCS28 (Supplementary Figure S2).

The constructs pBinGlyRed2-AhKCS1 and pBinGlyRed2-AhKCS28 were introduced into *Agrobacterium tumefaciens* GV3101 by electro-transformation. The *Arabidopsis fae1/fad2* mutant was transformed according to the previously described method (Zhang et al., 2015). DsRed-positive seeds were identified using a green LED flashlight with a red camera filter lens (Huai et al., 2015).

### Gas Chromatographic Analysis of Fatty Acid Compositions

For *Arabidopsis*, the DsRed positive mature seeds from each line were analyzed as a sample. For mature peanut seeds, 20 seeds from each line were collected and grinded as a sample. For developing peanut seeds, 5–10 seeds from each line were collected, lyophilized and grinded as a sample.

FAMEs were prepared from 25 mg finely grounded seeds with 2.5% (v/v) sulfuric acid/methanol as previously described (Huai et al., 2015). Fatty acids were transmethylated at 90°C for 1 h. After cooling to room temperature, 1 ml of aqueous 0.9% NaCl was added, and FAMEs were recovered by three sequential extractions with 1 ml of hexane. FAMEs were analyzed using an Agilent 7890B gas chromatograph with flame ionization detection and the DB-23 column. Fatty acids were identified by retention time according to previous studies (Huai et al., 2018; Liu et al., 2019).

### Statistical Analysis

For correlation analysis on expression levels of *AhKCS* genes and VLCFA contents, the expression level data of each *AhKCS* gene and the contents of VLCFAs were collected at the same developing stage. For association analysis between *AhKCS* expression level and VLCFA content in different lines, the expression level data of each *AhKCS* gene at stage III was correlated with the VLCFA content in mature seeds. Correlation coefficients were estimated using the IBM SPSS Statistics software. For the comparison of multiple means, the test for statistical significance was performed with ANOVA and Fisher’s least significant difference (LSD) multiple-comparison test, using the same software. In all the analyses, only *P* < 0.05 was considered to be significant.

## Results

### Identification of *AhKCS* Gene Family Members in the Peanut Genome

**FIGURE 1:**
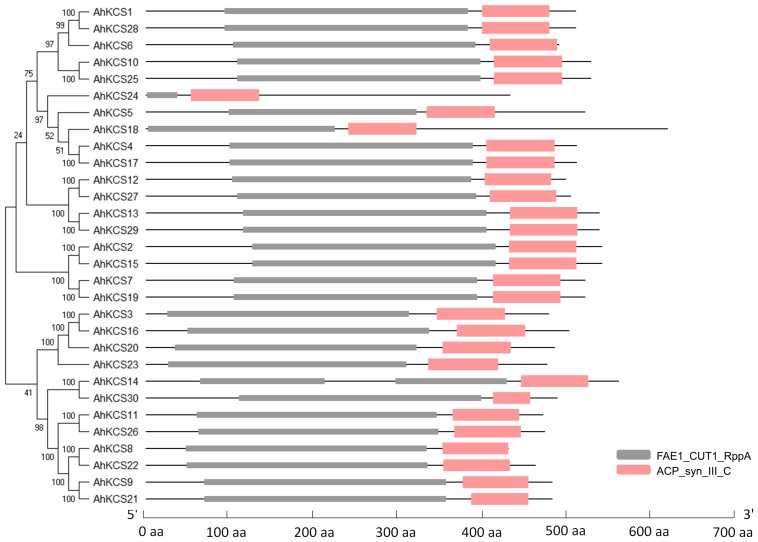
The conserved domains of AhKCSs from peanut. FAE1_CUT1_RppA: FAE1/Type III polyketide synthase-like protein domain (PF08392); ACP_syn_III_C: 3-Oxoacyl-[acyl-carrier-protein (ACP)] synthase III C terminal domain (PF08541).

To identify all the *AhKCS* genes in the genome of the peanut, HMMER and BLAST searches were performed using 21 *AtKCS* genes from *Arabidopsis* as the query (Supplementary Table S4). A total of 30 putative AhKCSs were identified in the peanut, and were numbered based on their chromosomal locations, respectively (Table 1). None of these putative *AhKCSs* showed significant homology to *AtELO* genes (Supplementary Table S5), indicating that all the identified *AhKCS* genes were FAE1-type. All AhKCSs contained two domains, a 3-Oxoacyl-[acyl-carrier-protein (ACP)] synthase III C terminal domain (ACP_syn_III_C) and a FAE1/Type III polyketide synthase-like protein domain (FAE1_CUT1_RppA) (Figure 1). The putative *AhKCS* genes encoded proteins ranging from 432 amino acids to 619 amino acids. Most of these genes contained less than four introns, while seven *AhKCS* genes harbored no intron throughout their whole open reading frames. The alternatively spliced transcripts were identified in *AhKCS14* and *AhKCS22* genes (Table 1), implying that different isoforms may play different roles in the development of the peanut.

**TABLE 1:** *AhKCS* genes identified in the peanut genome.

ID	Accession	Chr.*	No. of introns	CDS length (bp)	AA
*AhKCS1*	Arahy.IFJ1V3	01	0	1533	510
*AhKCS2*	Arahy.AYAJ7Z	02	2	1626	541
*AhKCS3*	Arahy.DFH8S5	03	1	1437	478
*AhKCS4*	Arahy.T9PLK1	03	0	1536	511
*AhKCS5*	Arahy.0744GE	04	4	1563	521
*AhKCS6*	Arahy.T43VF5	05	1	1473	490
*AhKCS7*	Arahy.LACR9M	06	0	1566	521
*AhKCS8*	Arahy.BRB394	07	2	1293	430
*AhKCS9*	Arahy.LP0WMI	07	1	1449	482
*AhKCS10*	Arahy.1FMC3R	09	1	1587	528
*AhKCS11*	Arahy.A9ZVJV	09	1	1416	471
*AhKCS12*	Arahy.N2G1VT	10	2	1497	498
*AhKCS13*	Arahy.WQ1I1V	10	2	1617	538
*AhKCS14*	Arahy.UB51SX	10	4 or 2	1686	561
*AhKCS15*	Arahy.06CD8W	12	2	1626	541
*AhKCS16*	Arahy.PB1D04	13	1	1509	502
*AhKCS17*	Arahy.TIY3DH	13	0	1536	511
*AhKCS18*	Arahy.V2BP3N	13	2	1860	619
*AhKCS19*	Arahy.V6AFRQ	16	0	1566	521
*AhKCS20*	Arahy.F7NA0C	18	0	1458	485
*AhKCS21*	Arahy.MXV0CY	18	1	1449	482
*AhKCS22*	Arahy.JWBY7T	18	2 or 1	1389	462
*AhKCS23*	Arahy.3ATP19	18	2	1431	476
*AhKCS24*	Arahy.7AH051	19	2	1299	432
*AhKCS25*	Arahy.XI5WK7	19	1	1587	528
*AhKCS26*	Arahy.VJNQ2E	19	1	1422	473
*AhKCS27*	Arahy.X66CUM	20	2	1515	504
*AhKCS28*	Arahy.BGR17W	20	0	1533	510
*AhKCS29*	Arahy.YW30D2	20	2	1617	538
*AhKCS30*	Arahy.A9N57E	20	2	1464	488

*Chromosomes 01–10 belong to the A subgenome; chromosomes 11–20 belong to the B subgenome.

**FIGURE 2:**
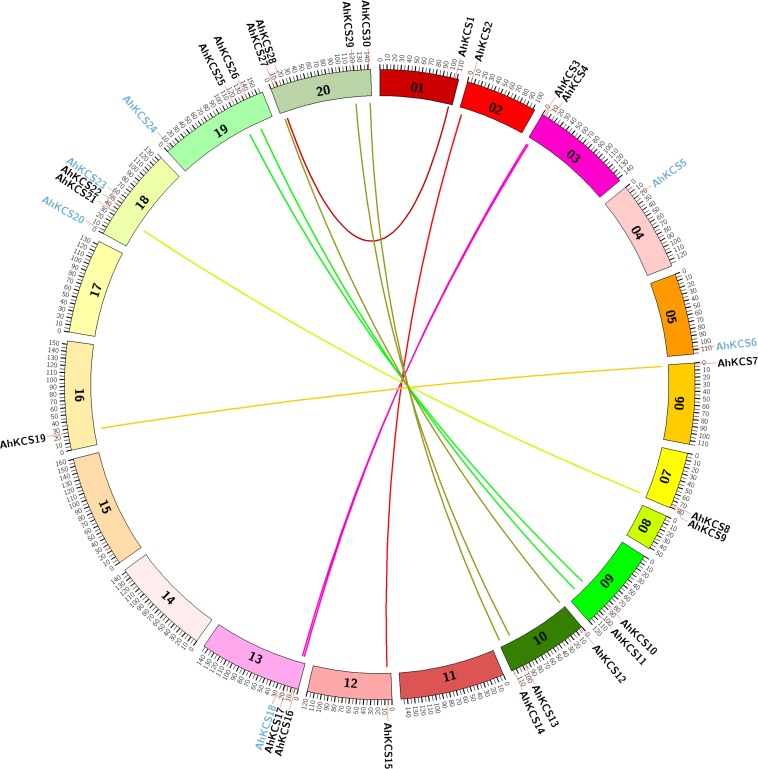
Genome-wide syntenic analysis of *AhKCS* genes on the chromosomes in peanut. 01–10, chromosomes from the A subgenome; 11–20, chromosomes from the B subgenome. The colored lines indicate the syntenic gene pairs between A and B subgenomes. The blue character represents the non-syntenic *KCS* genes.

*AhKCS* genes were widely distributed throughout genomes but were uneven among chromosomes. There were 14 *AhKCS* genes (*AhKCS1* – *AhKCS14*) located on nine chromosomes in subgenome A, and 16 *AhKCS* genes (*AhKCS15*-*AhKCS30*) on six chromosomes in subgenome B (Table 1 and Figure 2). No *AhKCS* gene was detected on chromosome 08 in subgenome A, as well as chromosomes 11, 14, 15, and 17 in subgenome B (Table 1 and Figure 2). More than two *AhKCS* genes were identified on chromosomes 10 (3), 13 (3), 18 (4), 19 (3), and 20 (4) (Table 1 and Figure 2). Three chromosomes, 03, 07, and 09 each harbored two *AhKCS* genes, while seven chromosomes, 01, 02, 04, 05, 06, 12, and 16 each had just one *AhKCS* gene (Table 1 and Figure 2).

Syntenic gene pairs are illustrated with a Circos diagram (Figure 2). Twelve *AhKCS* genes in subgenome A were paired with *AhKCS* genes in subgenome B and shared over 90% homology (Figure 2). *AhKCS* genes on chromosomes 02, 03, 06, 09, and 10 were syntenic to *AhKCS* genes on corresponding chromosomes 12, 13, 16, 19, and 20. However, *AhKCS1* on chromosome 01 was paired with *AhKCS28* on chromosome 20. Similarly, *AhKCS8* and *AhKCS9* on chromosome 07 were paired with *AhKCS21* and *AhKCS22* on chromosome 18 (Figure 2). *AhKCS5* and *AhKCS6* in subgenome A were not paired, as well as four *AhKCS* genes in subgenome B (*AhKCS18*, *AhKCS20*, *AhKCS23*, and *AhKCS24*) (Figure 2).

### Phylogenetic Analysis of AhKCSs From Peanut Plants

**FIGURE 3:**
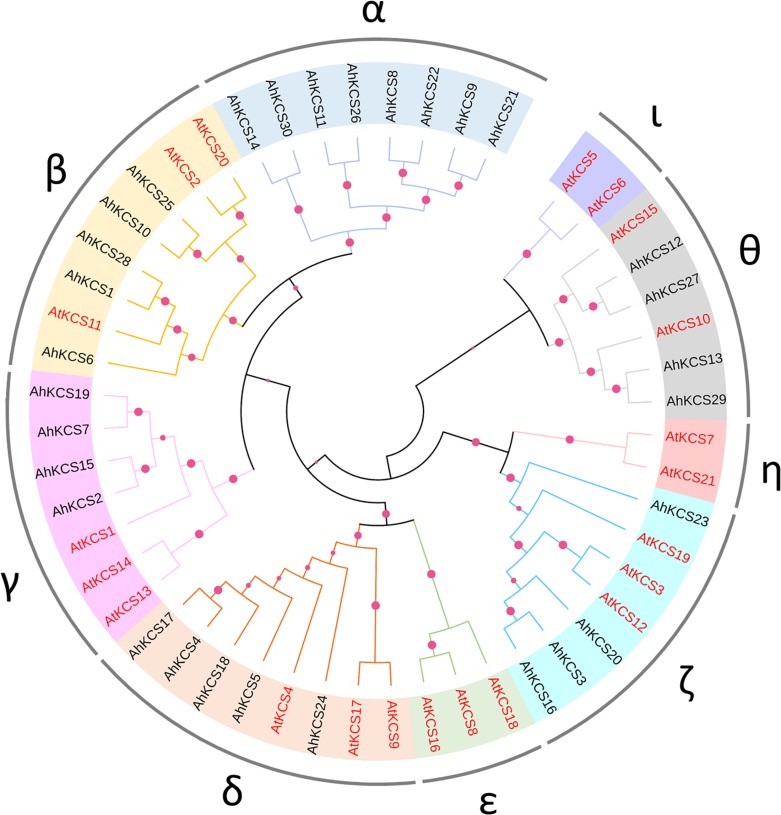
Phylogenetic analysis of AhKCS family members in peanut. A neighbor-joining phylogenetic tree was generated by MEGA X with full-length KCS sequences from the peanut and *Arabidopsis*. The colored circles at each node represent the bootstrap support (percentage).

An un-rooted phylogenetic tree was constructed in MEGA X based on the protein sequences of AhKCSs from the peanut, plus the *Arabidopsis* AtKCSs. The KCS proteins were divided into nine groups: α, β, γ, δ, ε, ζ, η, θ, and ι. No AhKCS was classified into Group ε, η, and ι, while no AtKCS was detected in Group α (Figure 3). These results indicate that *AhKCS* genes in the peanut might have undergone gene duplication and ensuing sub-functionalization and/or neo-functionalization, generating more complicated functions in the peanut than in *Arabidopsis*.

Group α contained eight AhKCSs: AhKCS8, AhKCS9, AhKCS11, AhKCS14, AhKCS21, AhKCS22, AhKCS26, and AhKCS30, which were not grouped with any AtKCS. Group β harbored five AhKCSs (AhKCS1, AhKCS6, AhKCS10, AhKCS25, and AhKCS28) and three AtKCSs including the AtKCS2/DAISY AtKCS11 and AtKCS20. There were four AhKCSs (AhKCS2, AhKCS7, AhKCS15, and AhKCS19) and three AtKCSs (AtKCS1, AtKCS13, and AtKCS14) in Group γ. Five AhKCSs (AhKCS4, AhKCS5, AhKCS17, AhKCS18, and AhKCS24) and three AtKCSs (AtKCS4, AtKCS9, and AtKCS17) were classified into Group δ. In Group ζ, 4 AhKCSs (AhKCS3, AhKCS16, AhKCS20, and AhKCS23) were grouped with three AtKCSs (AtKCS3, AtKCS12, and AtKCS19). In Group θ, four AhKCSs (AhKCS12, AhKCS13, AhKCS27, and AhKCS29) were closely related to AtKCS10/FDH and AtKCS15 (Figure 3).

It has been proved that the seed specific expressed AtKCS18/FAE1 controls the VLCFA content in seed of *Arabidopsis* (James et al., 1995), so the peanut ortholog which was grouped with AtKCS18/FAE1 was of interest. However, there was no AhKCS classified into Group ε with AtKCS18/FAE1 (Figure 3). This might imply that there was no seed-specific AhKCS that regulated VLCFA content only in the seed.

### Expression Patterns of *AhKCS* Genes in Peanut Plants

**FIGURE 4:**
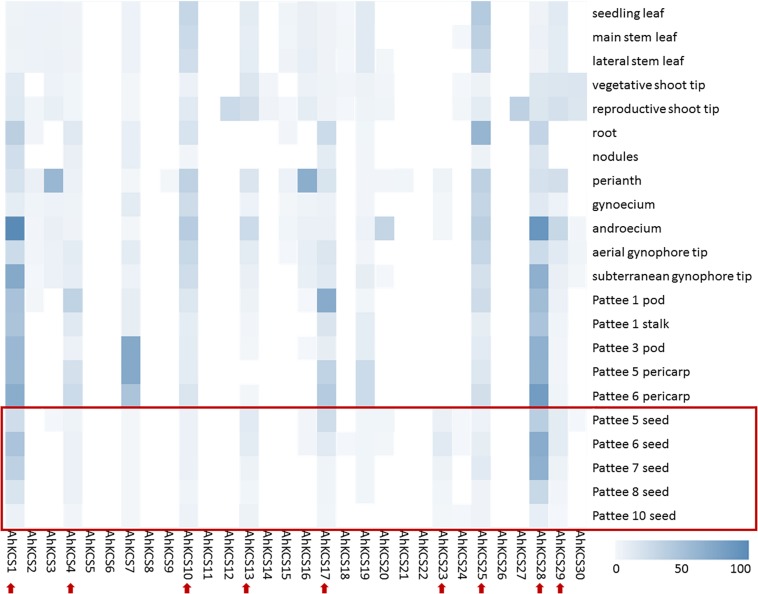
Heat map showing the expression patterns of *AhKCS* genes in 22 tissues of peanut. Seedling leaves and roots were collected 10 days post emergence; nodules were collected 25 days post emergence; main stem leaves, lateral leaves, vegetative shoot tips from main stem, and reproductive shoot tips from first lateral were collected when the first flower appeared; perianth, gynoecia and androecia were collected when flowers were blooming in the morning of anthesis; aerial gynophore tips were collected at elongating pegs prior to soil penetration; subterranean gynophore tips were collected 24 h after soil penetration; Pattee 1 pod, whole pod at pod swelling; Pattee 1 stalk, gynophore stalk at pod swelling; Pattee 3 pod, pericarp very watery, embryo very small and not easily removed; Pattee 5 pericarp, pericarp soft, not as watery, inner pericarp without cracks; Pattee 5 seed, embryo flat, white or just turning pink at one end; Pattee 6 pericarp, inner pericarp tissue beginning to show cracks or cottony; Pattee 6 seed, torpedo shaped, generally pink at embryonic-axis end of kernels; Pattee 7 seed, torpedo to round shaped, embryonic axis end of kernel pink, other end white to light pink; Pattee 8 seed, round, light pink all over; Pattee 10 seed, large, generally dark pink all over, seed coat beginning to dry out. Red arrows indicate *AhKCS* genes with some expression level in developing seed. The data was downloaded from NCBI (Clevenger et al., 2016).

The expression levels of *AhKCS* genes in 22 tissues of the peanut were investigated using transcriptome data to identify those with the highest expression in the developing seed (Clevenger et al., 2016). The FPKM values of *AhKCS* genes were calculated by mapping reads to the peanut genome. A heat-map of *AhKCS* genes was created to demonstrate their expression profile (Figure 4).

In total, the expression of 22 *AhKCS* genes was detected in at least one tissue of the peanut (Figure 4). Six *AhKCS* genes (*AhKCS5*, *AhKCS6*, *AhKCS8*, *AhKCS11*, *AhKCS22*, and *AhKCS26*) were not expressed in any of the tested tissues from which the transcriptome data were derived (Figure 4). Eleven *AhKCS* genes (*AhKCS1*, *AhKCS2*, *AhKCS4*, *AhKCS7*, *AhKCS10*, *AhKCS13*, *AhKCS17*, *AhKCS19*, *AhKCS25*, *AhKCS28*, and *AhKCS29*) were constitutively expressed in the 22 tissues (Figure 4). *AhKCS12* and *AhKCS27* were specifically expressed in the leaf and stem, while *AhKCS3*, *AhKCS16*, and *AhKCS20* were specifically expressed in the flower (Figure 4). No *AhKCS* gene was specifically expressed in the roots or gynophores (Figure 4). None of the *AhKCS* genes showed seed-specific expression, though *AhKCS23* was dominantly expressed in both developing seeds and flowers (Figure 4).

Nine *AhKCS* genes, namely, *AhKCS1*, *AhKCS4*, *AhKCS10*, *AhKCS13*, *AhKCS17*, *AhKCS23*, *AhKCS25*, *AhKCS28*, and *AhKCS29*, were expressed in developing seeds (Figure 4). Except for *AhKCS23*, the other genes were constitutively expressed (Figure 4). From these results, candidate genes that might regulate VLCFA content in the developing peanut seed were selected for further characterization.

### Correlation Analyses on Expression Levels of *AhKCS* and Contents of VLCFAs

**FIGURE 5:**
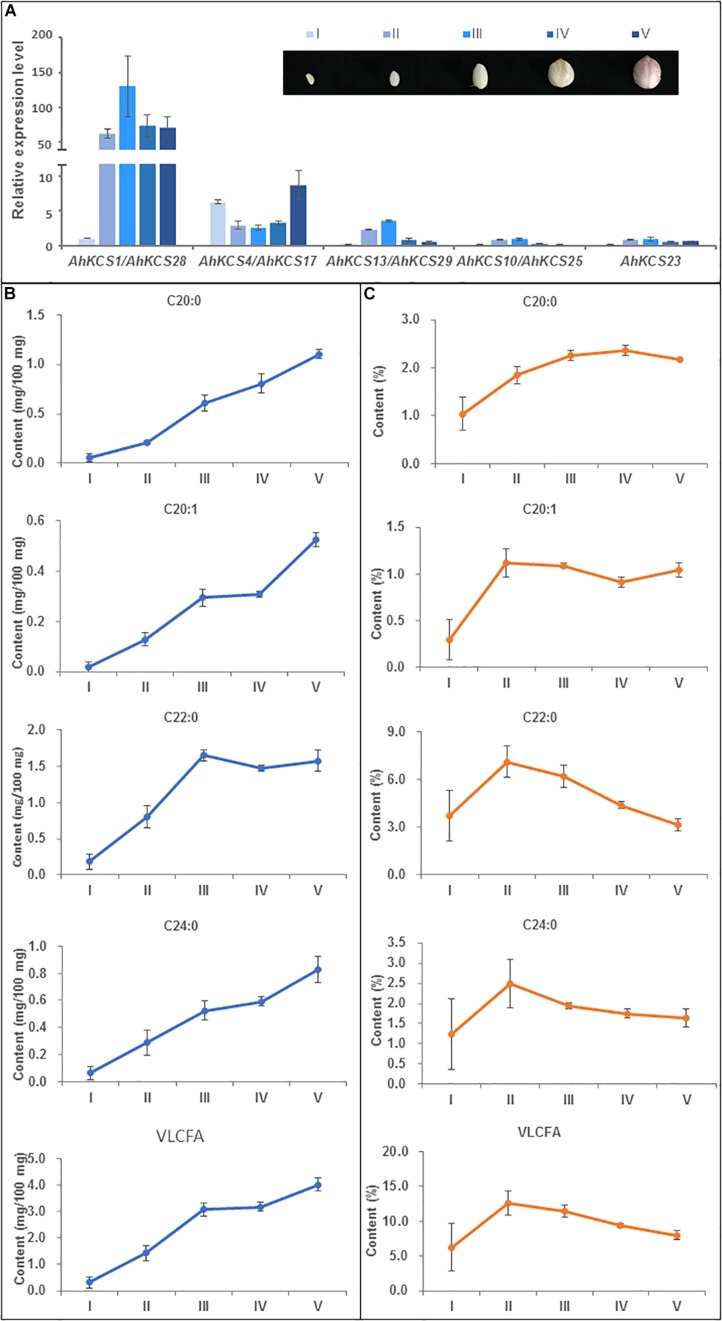
Relationship between *AhKCS* gene expression and the accumulation of VLCFAs during peanut seed development. **(A)** qRT-PCR analysis of *AhKCS* genes in developing peanut seeds. **(B)** Absolute contents of VLCFAs during peanut seed development. **(C)** Relative contents of VLCFAs during peanut seed development. Developing peanut seeds were harvested at five specific stages: I – white and flat embryo [approximately 20 days after pollination (DAP)]; II – white and teardrop-shaped embryo (30 DAP); III – white and torpedo to round shaped embryo (40 DAP); IV – light pink and round embryo (50 DAP); V – dark pink, large, and round embryo (60 DAP). *AhACTIN* gene expression level was used as a constitutive control. Data are presented as means with standard error bars SD, which were calculated based on 3–4 independent replicates. Samples from each replicate were measured using 5–10 developing seeds. Statistical significance was evaluated by ANOVA and Fisher’s least significant difference (LSD) multiple comparison.

Analysis of VLCFA content in developing seeds of Zhonghua16 were conducted at 20, 30, 40, 50, and 60 DAP. During the development of the seed, no decrease was observed in the accumulation of VLCFAs, except for C22:0 (Figure 5B). The contents of C20:0 and C24:0 continuously increased during the tested developmental stages (Figure 5B). The content of C20:1 soared during 20–40 DAP, plateaued during 40–50 DAP, and then sharply increased during 50–60 DAP (Figure 5B). However, the content of C22:0 sharply increased during 20–40 DAP, and then slightly decreased during 40-50 DAP followed by a moderate increase in the last 10 days (Figure 5B). The total content of VLCFA increased during 20–40 DAP, plateaued during 40–50 DAP, and then continued to increase (Figure 5B).

Nevertheless, the relative contents of VLCFA first increased and then decreased during seed development (Figure 5C). The relative content of C20:0 gradually increased to a peak at 50 DAP, while the relative content of other VLCFAs, including total content of VLCFA, dramatically rose to a peak at 30 DAP (Figure 5C), suggesting that the relative contents of VLCFAs in the mature seed were lower.

*AhKCS* gene expression levels in developing peanut seeds were confirmed by qPCR at the same developing stages. Except for *AhKCS4* and *AhKCS17*, the expression levels of other *AhKCS* genes rapidly increased during 20–40 DAP, and then slightly decreased during 40–60 DAP (Figure 5A). In contrast, the expression level of *AhKCS4* and *AhKCS17* first decreased during 20–40 DAP, then increased during 40–60 DAP (Figure 5A). The expression level of *AhKCS1* and *AhKCS28* were 5–10-fold higher than that of any other *AhKCS* gene in all developing stages except for stage I (Figure 5A). In later stages of development, no significant difference among the expression levels of the remaining *AhKCS* genes was detected, except for *AhKCS1* and *AhKCS28* (Figure 5A). These results confirmed that the expression patterns of *AhKCS* genes by qPCR analysis (Figure 5A) were consistent with transcriptome data (Figure 4).

The correlation analysis on expression levels of *AhKCS* genes and VLCFA contents was conducted. Only the expression levels of *AhKCS1* and *AhKCS28* were significantly and positively correlated with the content of C22:0 (*R*^2^ = 0.90, *p* < 0.05) (Supplementary Table S6). Unfortunately, the expression levels of other *AhKCS* genes were not correlated with any content of VLCFA (Supplementary Table S6). These results implied that among these nine *AhKCS* genes, *AhKCS1*, and *AhKCS28* are the best candidate contributors to the accumulation of VLCFA in the peanut seed.

### Heterologous Expression of *AhKCS* Genes in Yeast

To analyze the functions of the proteins encoded by the *AhKCS* genes, the coding regions of each gene was inserted to the vector pYX242 and transformed into yeast, along with *BnaA.FAE1* and *BnaC.FAE1* genes from rapeseed (*Brassic napus* L.) (Huai et al., 2018) as controls. Fatty acid profiles of the induced yeast cell were obtained as the evidence of substrate preference.

**FIGURE 6:**
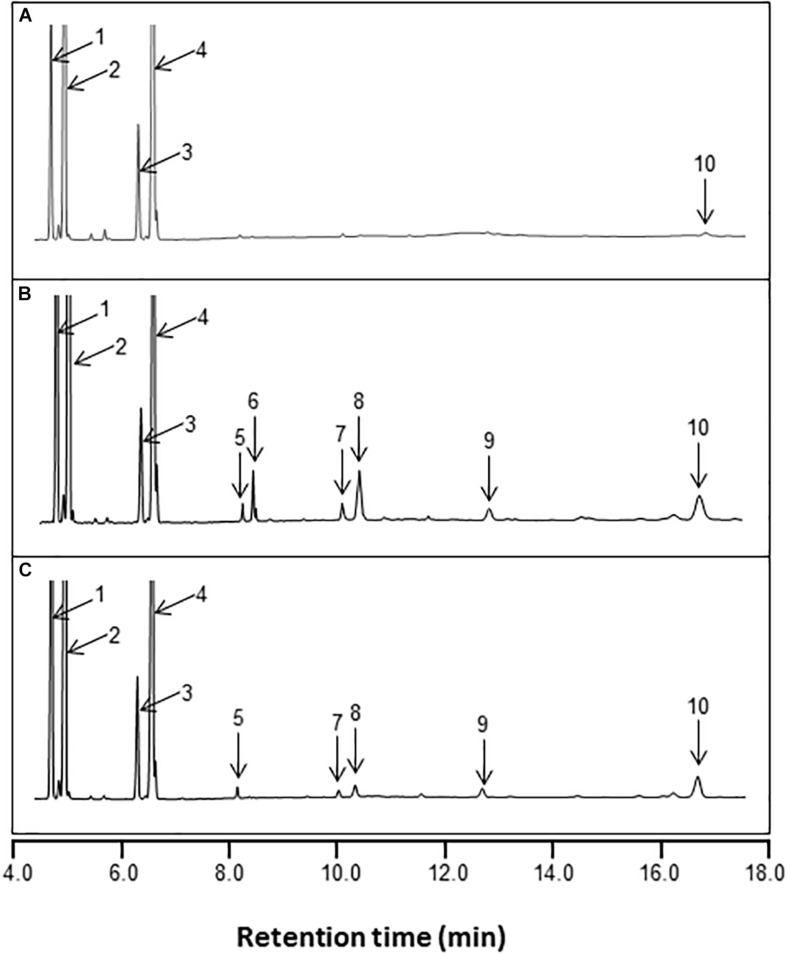
GC analysis of fatty acid methyl esters (FAMEs) from the total lipid fraction of yeast transformed with pYX242 **(A)**, pYX242-BnaA.FAE1 **(B),** and pYX242-AhKCS1 **(C)**. Fatty acid peak identifies are: 1 = C16:0; 2 = C16:1Δ9; 3 = C18:0; 4 = C18:1Δ9 and C18:1Δ11; 5 = C20:0; 6 = C20:1Δ11; 7 = C22:0; 8 = C22:1Δ13; 9 = C24:0; 10 = C26:0. C16:0, palmitic acid; C16:1, palmitoleic acid; C18:0, stearic acid; C18:1, oleic acid; C20:0, arachidic acid; C20:1, eicosenoic acid; C22:0, behenic acid; C22:1, erucic acid; C24:0, lignoceric acid; C26:0, hexacosanoic acid.

New peaks of saturated and monounsaturated VLCFAs, which are normally present in low abundance in yeast, were detected in yeast cells expressing *AhKCS* genes, and the VLCFA content in these cells was significantly increased (Figure 6 and Table 2). This result indicated that all the tested AhKCSs possessed fatty acid elongase activities. However, the VLCFA compositions in yeast cells with *AhKCS* genes were not the same. Five peaks for C20:0, C22:0, C22:1, C24:0, and C26:0 were detected in yeast cells with *AhKCS1*, *AhKCS10*, *AhKCS23*, *AhKCS25*, and *AhKCS28* genes, while only two peaks for C20:0 and C26:0 were detected in yeast cells with *AhKCS13* and *AhKCS29* (Table 2). There were four peaks for C20:0, C22:0, C22:1, and C26:0 identified in yeast cells with *AhKCS4* and *AhKCS17* (Table 2). These results suggested that the substrate specificities of AhKCSs differed: AhKCS13 and AhKCS29 tended to accumulate C20:0; AhKCS4 and AhKCS17 preferentially stored C26 fatty acids; AhKCS1, AhKCS10, AhKCS23, AhKCS25, and AhKCS28 actively produced a variety of VLCFAs.

**TABLE 2:** VLCFA contents (%) in yeast transformed with empty vector and *AhKCS* genes.

Construct	C20:0	C20:1	C22:0	C22:1	C24:0	C26:0	VLCFA	VLCSFA	VLCUFA	VLCSFA/VLCUFA
pYX242	ND	ND	ND	ND	ND	0.4 ± 0.1^b^	0.4 ± 0.1^e^	0.4 ± 0.1^e^	ND	
pYX242-BnaA.FAE1	0.5 ± 0.1^b^	1.1 ± 0.1^b^	0.9 ± 0.2^a^	3.0 ± 1.1^b^	1.5 ± 0.3^a^	0.3 ± 0.2^b^	7.4 ± 1.6^ab^	3.2 ± 0.2^bc^	4.1 ± 1.2^b^	0.9 ± 0.3^b^
pYX242-BnaC.FAE1	0.4 ± 0.1^b^	1.4 ± 0.1^a^	0.7 ± 0.1^ab^	4.3 ± 0.5^a^	1.5 ± 0.7^a^	0.2 ± 0.1^b^	8.5 ± 1.2^a^	2.7 ± 0.9^cd^	5.8 ± 0.5^a^	0.5 ± 0.1^b^
pYX242-AhKCS1	0.5 ± 0.1^b^	ND	0.4 ± 0.1^bc^	0.6 ± 0.2^c^	0.6 ± 0.1^ab^	3.3 ± 0.6^a^	5.4 ± 0.6^bc^	4.8 ± 0.8^ab^	0.6 ± 0.2^c^	9.8 ± 4.1^a^
pYX242-AhKCS4	0.2 ± 0.1^b^	ND	0.1 ± 0.0^c^	0.5 ± 0.2^c^	ND	2.8 ± 0.7^a^	3.6 ± 1.0^cd^	3.1 ± 0.8^bcd^	0.5 ± 0.2^c^	7.4 ± 2.3^a^
pYX242-AhKCS10	0.1 ± 0.0^b^	ND	0.2 ± 0.1^c^	0.1 ± 0.1^c^	0.2 ± 0.1^b^	1.0 ± 0.2^b^	1.5 ± 0.3^d^	1.4 ± 0.2^d^	0.1 ± 0.1^c^	12.7 ± 5.7^a^
pYX242-AhKCS13	2.5 ± 0.9^a^	ND	ND	ND	ND	0.6 ± 0.2^b^	3.1 ± 0.8^d^	3.1 ± 0.8^bcd^	ND	
pYX242-AhKCS17	0.3 ± 0.1^b^	ND	0.2 ± 0.0^c^	0.5 ± 0.1^c^	ND	3.6 ± 0.4^a^	4.3 ± 0.6^c^	3.9 ± 0.5^abc^	0.5 ± 0.1^c^	8.5 ± 2.7^a^
pYX242-AhKCS23	0.5 ± 0.1^b^	ND	0.5 ± 0.2^b^	0.7 ± 0.2^c^	0.9 ± 0.3^ab^	3.0 ± 0.2^a^	5.6 ± 0.9^b^	4.9 ± 0.8^ab^	0.7 ± 0.2^c^	7.0 ± 1.0^a^
pYX242-AhKCS25	0.3 ± 0.1^b^	ND	0.4 ± 0.0^c^	0.6 ± 0.1^c^	0.8 ± 0.2^ab^	0.9 ± 0.3^b^	3.0 ± 0.7^d^	2.4 ± 0.6^cd^	0.6 ± 0.1^c^	5.8 ± 2.4^a^
pYX242-AhKCS28	0.4 ± 0.1^b^	ND	0.5 ± 0.2^bc^	0.7 ± 0.3^c^	0.9 ± 0.3^ab^	3.7 ± 0.4^a^	6.1 ± 0.9^b^	5.4 ± 0.4^ab^	0.7 ± 0.3^c^	9.6 ± 3.3^a^
pYX242-AhKCS29	3.1 ± 1.3^a^	ND	ND	ND	ND	0.8 ± 0.3^b^	4.0 ± 1.1^d^	4.0 ± 1.1^bcd^	ND	

**ND, not detected. C20:0: arachidic acid; C20:1: eicosenoic acid; C22:0: behenic acid; C22:1: erucic acid; C24:0: lignoceric acid; C26:0: hexacosanoic acid. VLCFA = C20:0 + C20:1 + C22:0 + C22:1 + C24:0 + C26:0; VLCSFA = C20:0 + C22:0 + C24:0 + C26:0; VLCUFA = C20:1 + C22:1. Means ± SD were calculated based on 3–5 independent replicates. a, b, c, d, and e in each column represent significant difference at P < 0.05 based on ANOVA and Fisher’s least significant difference (LSD) multiple-comparison. Each letter is significantly different from any other letter and any combination of other letters but is not significantly different from itself and any combination of these letters including itself. For example, a is significantly different from b, c, d, e, bc, cd, de, bcd, cde, and bcde, but is not significantly different from a, ab, abc and abcd. ab is significantly different from c, d, e, cd, de, and cde, but is not significantly different from a, b, ab, abc and abcd. abc is significantly different from d, e and de, but is not significantly different from a, b, c, ab, bc, abc and abcd. abcd is significantly different from e, but is not significantly different from a, b, c, d, ab, bc, cd, abc, bcd, and abcd. The same for b, c, d, and e.**

Compared to BnaA.FAE1 and BnaC.FAE1 from rapeseed, AhKCSs exhibited higher preference for C16 and/or C18 SFAs as substrates. In yeast cells expressing *BnaA.FAE1* or *BnaC.FAE1*, C20:1 and C22:1 were detected but only C22:1 was found in *AhKCSs*-expressing cells (Figure 6 and Table 2). The content of very long-chain unsaturated fatty acids (VLCUFAs; C20:1 + C22:1) was significantly higher than that of very long-chain saturated fatty acids (VLCSFAs; C20:0 + C22:0 + C24:0 + C26:0) in yeast cells expressing *BnaC.FAE1* (*P* < 0.05), though no obvious difference was observed in yeast cells expressing *BnaA.FAE1*. Conversely, the content of VLCUFA was significantly lower than that of VLCSFA in all yeast cells expressing any *AhKCS* gene (*P* < 0.05). Additionally, the ratio of VLCSFA to VLCUFA in yeast cells expressing BnaA.FAE1 or BnaC.FAE1 were 0.9 and 0.5, respectively, while the ratios in all yeast cells expressing any *AhKCS* gene were greater than 5.0 (Table 2). These results indicated that AhKCSs were more specific to saturated fatty acid substrates than Bna.FAE1s.

### Analyses of AhKCS Genes in Peanut Lines With Different Contents of VLCFA

**FIGURE 7:**
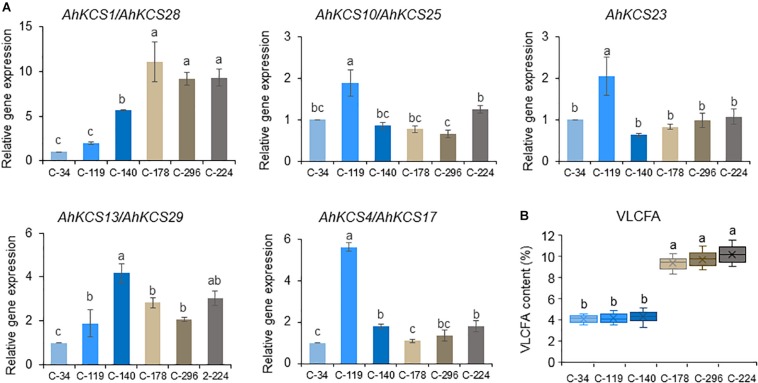
Expression levels of *AhKCS* genes in developing seeds from peanut lines with different VLCFA contents. **(A)** qRT-PCR analysis of *AhKCS* genes in developing seeds from peanut lines with different VLCFA contents. **(B)** VLCFA contents in mature seeds harvested from C-34, C-119, C-140, C-178, C-296, and C-224. The relative expression values of genes were measured on developing seeds harvested at 50 days after pollination. *AhACTIN* gene expression level was used as a constitutive control. Values are the means and SD of three independent biological replicates. a, b, and c represent significant difference at *P* < 0.05 based on ANOVA and Fisher’s least significant difference (LSD) multiple-comparison. Each letter is significantly different from any other letter and any combination of other letters but is not significantly different from itself and any combination of these letters including itself. For example, a is significantly different from b, c and bc, but is not significantly different from a and ab. b is significantly different from a and c, but is not significantly different from b, ab, and bc. c is significantly different from a, b, and ab, but is not significantly different from c and bc. ab is significantly different from c, but is not significantly different from a and b. bc is significantly different from a, but is not significantly different from b and c.

To confirm the roles of AhKCSs in regulating the VLCFA content of the peanut, these nine *AhKCS* genes were isolated and sequenced from six lines with different VLCFA contents in their seeds. The VLCFA contents in C-34, C-119, and C-140 were 4.6, 4.3, and 4.7%, while the VLCFA contents in C-178, C-296, and C-224 were 9.4, 9.6, and 9.8% (Figure 7B and Supplementary Table S7). However, no single nucleotide polymorphism (SNP) was detected in the coding sequences of *AhKCS1*, *AhKCS4*, *AhKCS10*, *AhKCS13*, *AhKCS25*, *AhKCS28*, and *AhKCS29* among these six lines. One nucleotide substitution (C:G → A:T) at position 39 was identified in *AhKCS17* of C-119, with no amino acid alternation (Supplementary Figure S3A). Another nucleotide substation (T:A → C:G) at position 1330 was identified in *AhKCS23* of C-34 without changing the amino acid (Supplementary Figure S3B). None of the SNPs were therefore associated with VLCFA content.

The expression levels of AhKCS genes were further investigated in the developing seeds from these six lines (Figure 7). All nine *AhKCS* genes were expressed, but only the expression levels of *AhKCS1* and *AhKCS28* were positively correlated with the VLCFA content (*R*^2^ = 0.93, *p* < 0.05) (Supplementary Table S8). The expression levels of *AhKCS1* and *AhKCS28* in C-34, C-119, and C-140 were at least twice as low as those in C-178, C-296, and C-224 (Figure 7A). There was no significant correlation between the expression levels of other *AhKCS* genes and the seed VLCFA content (Figure 7 and Supplementary Table S8). Based on these results, *AhKCS1* and *AhKCS28* were proposed to regulate the VLCFA content in peanut.

### Subcellular Localization of AhKCS1 and AhKCS28

**FIGURE 8:**
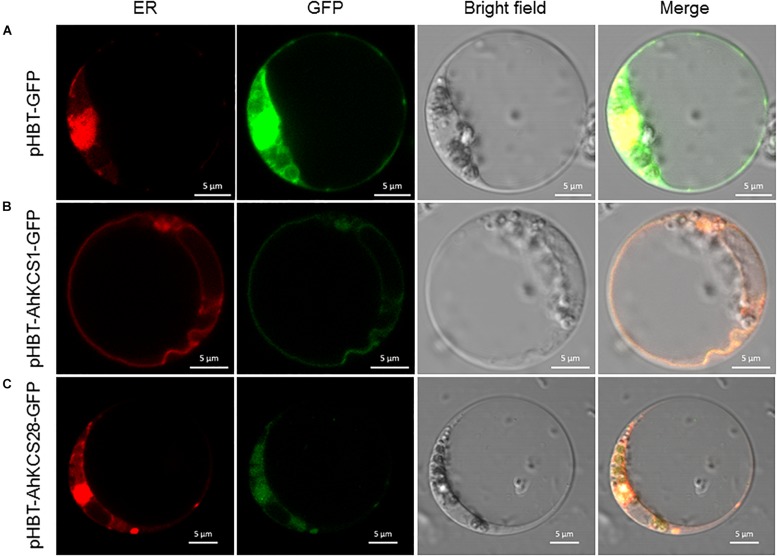
Subcellular location of AhKCS1 and AhKCS28 in the *Arabidopsis* protoplast cell. **(A)** An empty GFP vector served as the control. **(B)** AhKCS1-GFP (green) merged with endoplasmic reticulum (ER) marker (red). **(C)** AhKCS28-GFP (green) merged with ER marker (red).

To examine the subcellular localization of AhKCS1 and AhKCS28, they were separately fused with GFP, and then co-expressed with the ER marker in *Arabidopsis* protoplasts by PEG induction. As expected, the green signal of the empty vector spread throughout the whole cell (Figure 8A). But the green signal of AhKCS1 (Figure 8B) and AhKCS28 (Figure 8C) were completely co-localized with the red ER marker, respectively. No green signal was observed in the nucleus (Figures 8B,C). These results indicated that AhKCS1 and AhKCS28 were located on the ER, where fatty acid elongase complex functions (Haslam and Kunst, 2013).

### Heterologous Expression of *AhKCS1* and *AhKCS28* Genes in *Arabidopsis*

To further confirm the function of AhKCS1 and AhKCS28 in plants, the *AhKCS1* and *AhKCS28* genes were heterologous expressed in the *Arabidopsis fae1/fad2* double mutant. Eight *AhKCS1*-expressing T_1_ lines and five *AhKCS28*-expressing T_1_ lines were obtained, respectively (Supplementary Table S9). The fatty acid composition of the DsRed positive seeds from each line was determined. The VLCFA contents in seeds from the *Arabidopsis fae1/fad2* double mutant with *BnaA.FAE1* or *BnaC.FAE1* (Huai et al., 2018) were also used as controls to compare the substrate specificity.

**FIGURE 9:**
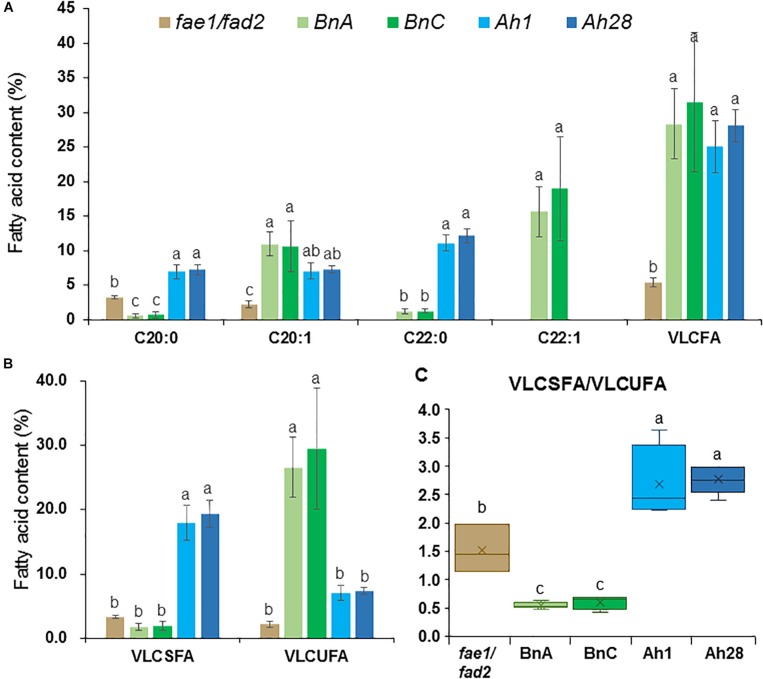
The VLCFA content in mature seeds harvested from *Arabidopsis fae1/fad2* double mutant and T_2_ transgenic lines. **(A)** The contents of C20:0, C20:1, C22:0, C22:1 and VLCFA. **(B)** The contents of very long chain saturated fatty acid (VLCSFA) and very long chain unsaturated fatty acid (VLCUFA). **(C)** The ratio of VLCSFA to VLCUFA. VLCFA = C20:0 + C20:1 + C22:0 + C22:1; VLCSFA = C20:0 + C22:0; VLCUFA = C20:1 + C22:1. BnA, T_2_ lines transformed with *BnaA.FAE1*; BnC, T_2_ lines transformed with *BnaC.FAE1*; Ah1, T_2_ lines transformed with *AhKCS1*; Ah28, T_2_ lines transformed with *AhKCS28*. At least three replicates were used for each sample. Samples from each replicate were measured using 25 mg mature seeds. Different letters indicated statistically different at *P* < 0.05 based on ANOVA and Fisher’s least significant difference (LSD) multiple-comparison. The data was shown in Supplementary Table S9.

In seeds of the *Arabidopsis fae1/fad2* double mutant, there were 3.2% of C20:0 and 2.2% of C20:1, without other VLCFAs (Figure 9A and Supplementary Table S9). In the seeds from *AhKCS1*- or *AhKCS28*-expressing lines, an extra VLCFA C22:0 was detected, and the contents of C20:0 and C20:1 were significantly increased. Moreover, the total VLCFA content was significantly increased from 5.5 to 25.8% and 28.4% in the *AhKCS1*- or *AhKCS28*-expressing lines, respectively (Figure 9A and Supplementary Table S9). These results suggested that AhKCS1 and AhKCS28 catalyzed fatty acid elongase activity in plants.

The compositions of VLCFAs in the seeds from *AhKCS1*- or *AhKCS28*-expressing lines were different to those in the seeds from *BnaA.FAE1*- and *BnaC.FAE1*-expressing lines (Figure 9A and Supplementary Table S9). In the *BnaA.FAE1*- and *BnaC.FAE1*-expressing lines, two extra VLCFAs were presented such as C22:0 and C22:1, compared to the VLCFAs in the *fae1/fad2* double mutant. C22:1 was absent in the seeds from the *AhKCS1*- or *AhKCS28*-expressing lines (Figure 9A). The C20:0 content was significantly increased from 3.2 to 6.9% in *AhKCS1*- and 7.2% in *AhKCS28*-expressing lines, while it was significantly decreased to 0.6% in *BnaA.FAE1*- and 0.7% in *BnaC.FAE1*-expressing lines (0.7%) (Figure 9A). In addition, there were more VLCSFAs and less VLCUFA accumulated in *AhKCS1*- or *AhKCS28*-expressing lines compared to *BnaA.FAE1*- or *BnaC.FAE1*-expressing lines (Figure 9B). Furthermore, the VLCSFA/VLCUFA ratio in *AhKCS1*- and *AhKCS28*-expressing lines were 2.6 and 2.8, but the ratios in *BnaA.FAE1*- and in *BnaC.FAE1*-expressing lines were 0.5 and 0.6 (Figure 9C). These results again confirmed that AhKCS1 and AhKCS28 were inclined to use saturated fatty acids as the substrate.

## Discussion

The high content of SFAs (15.5–27.5%) is a major negative quality factor in peanut oil. In the peanut, VLCSFA content varies from 2.5 to 8.5%, which accounts for 20–40% of the SFA content. Therefore, decreasing the VLCSFA content is an effective way to reduce SFA total content. It has been proved that the VLCFA content is mainly controlled by KCS in plants (James et al., 1995; Joubes et al., 2008; Huai et al., 2015), so we identified 30 *AhKCS* genes in the peanut genome (Table 1). There were approximately 20-30 members of the *KCS* gene family in angiosperms, such as 21 in *Arabidopsis*, 27 in soybean, and 19 in *Medicago truncatula* (Guo et al., 2016) – consistent with the large numbers of AhKCS family members in the peanut. All the identified AhKCSs contained two domains: an ACP_syn_III_C domain and a FAE1_CUT1_RppA domain, which have been proven to be necessary for KCSs (Figure 1; Sagar et al., 2015; Guo et al., 2016; Xiao et al., 2016). The ACP_syn_III_C domain is found in 3-Oxoacyl-[acyl-carrier-protein (ACP)] synthase III (EC:2.3.1.41), and is responsible for initiating the chain of fatty acid synthase reactions in plants and bacteria (Abbadi et al., 2000). KCS members harboring the FAE1_CUT1_RppA domain are described as 3-ketoacyl-CoA synthases, type III polyketide synthases, fatty acid elongases or fatty acid condensing enzymes, and are found in both prokaryotic and eukaryotic (mainly plant) species. This domain contains active site residues, as well as motifs involved in substrate binding (Nobutaka et al., 2002). The characteristics of ACP_syn_III_C and FAE1_CUT1_RppA domains supported that the genes identified in this study belong to *KCS* gene families.

Based on the phylogenetic analysis, all the AtKCS proteins were classified into eight groups (β, γ, δ, ε, ζ, η, θ, and ι) (Figure 3), which is consistent with previous studies (Joubes et al., 2008). All the identified AhKCS proteins were grouped into six groups: α, β, γ, δ, ζ, and θ (Figure 3). The AhKCSs in Group α were not grouped with any AtKCS (Figure 3), suggesting that these AhKCSs might be specific and acquired a different function in the peanut. The AhKCSs in group β were closely related to AtKCS2 and AtKCS20 (Figure 3), which were involved in the biosynthesis of suberin (Lee et al., 2009). The AhKCSs in group θ were grouped with AtKCS10 and AtKCS15 (Figure 3), which were reported to have participated in the biosynthesis of VLCFA in the epidermal cell (Pruitt et al., 2000). The AhKCSs in these two groups might also be involved in the development of the epidermis. Group ι was composed of AtKCS5/CER60 and AtKCS6/Cer6 only (Figure 3), which were responsible for the biosynthesis of cuticular wax (Fiebig et al., 2000). It hinted that the original homolog of AtKCS5/CER60 and AtKCS6/Cer6 might be deleted or functionally diverged in the peanut.

It has been shown that the VLCFA content in the seed is controlled by seed-specific expressed *KCS* genes, such as *AtFAE1* in *Arabidopsis* (James et al., 1995), *BnFAE1* in *B. napus* (Wang et al., 2010), and *CaFAE1* in *Crambe abyssinica* (Mietkiewska et al., 2007). However, no seed-specific expressing *AhKCS* gene was found in the peanut (Figure 4). Furthermore, no AhKCS was grouped with AtKCS18/FAE1 (Figure 3). Perhaps because the VLCFAs in the peanut are not as abundant as those in *Brassicaceae*, the seed-specific *AhKCS* genes eliminated during evolution.

*AhKCS1* and *AhKCS28* were considered to be the genes that regulate the content of VLCFA in the peanut for three reasons. First, the expression level of these two genes was the highest in developing seed (Figure 5A), suggesting that these two genes could be one of the main factors to regulate VLCFA biosynthesis in the peanut seed. Second, *AhKCS1* and *AhKCS28* were the only two genes, whose expression level was significantly and positively correlated with the VLCFA content (Figure 7A and Supplementary Table S8), indicating that the expression level of these two genes might affect the VLCFA content in the peanut seed. Third, AhKCS1 and AhKCS28 exhibited substrate preference for SFA, which were different from BnFAE1s (Table 2 and Figure 9). The substrate specificities are in accordance with the features of VLCFA composition in the peanut and rapeseed, showing that the substrate specificities of these two AhKCSs were involved in determining the VLCFA compositions in the peanut seed. In summary, AhKCS1 and AhKCS28 could be candidate genes for decreasing VLCFA content in the peanut seed.

Due to the high identity of the syntenic AhKCS pairs, the expression level detected in this study is the sum of both genes. For example, the expression level of *AhKCS1/AhKCS28* represented the total amount of expression levels of *AhKCS1* and *AhKCS28*. According to the expression profile of *AhKCS1* and *AhKCS2*8, both of them were expressed in the developing seed (Figure 4). But the homology between *AhKCS1*and *AhKCS28* was 97.8%, and there were only 14 SNPs between these two genes (Supplementary Figure S4). It is difficult to distinguish the expression levels from each other by qPCR. We designed five pairs of SNP specific primers, but we still failed to separate the expression levels between these two genes (data not shown). Although we could not figure out whose expression level decreased between *AhKCS1* and *AhKCS28*, we still proved that both were involved in the biosynthesis of VLCFAs in the peanut, since both *AhKCS1* and *AhKCS28* were highly expressed in the developing seed and possessed the fatty acid elongase activities.

Many studies have demonstrated that knocking out the *KCS* genes with a high expression level in seeds could significantly reduce the VLCFAs. For example, the VLCFA contents were dramatically decreased from >20% to <1% in the *Arabidopsis fae1* mutant (James et al., 1995), and from >40% to <2% in canola, which is the fae1 mutant of *B. napus* (Wang et al., 2010). The *fae1* mutants of *Camelina sativa* were created by the CRISPR/Cas9 technology, and the VLCFA content was reduced from 22 to <2% (Ozseyhan et al., 2018). Suppressing the expression level of *KCS* genes in seeds could also decrease the VLCFA content. When the expression of *FAE1* was silenced by RNAi in seeds of *B.napus*, the erucic acid was undetectable (Peng et al., 2010; Tian et al., 2011; Shi et al., 2017). Down-regulation of *FAE1* genes in *Crambe abyssinica* significantly reduced the erucic acid content (Li et al., 2016). Therefore, knockout or silencing of the *AhKCS1* and *AhKCS28* genes in the peanut might be an effective way to decrease the VLCFA content and improve the health-promoting and nutritional qualities of the peanut.

## Data Availability Statement

All datasets generated for this study are included in the article/Supplementary Material.

## Author Contributions

DH, YLei, and BL conceived and designed the experiments. HJ supplied the peanut lines. XX, JL, LY, YC, XW, NL, YK, and ZW performed the experiments. DH, YLi, PW, and YH analyzed the data. DH wrote the manuscript. DH, HJ, YLei, and BL revised the manuscript. All authors read and approved the final version of the manuscript.

## Conflict of Interest

The authors declare that the research was conducted in the absence of any commercial or financial relationships that could be construed as a potential conflict of interest.

## Footnotes

1 https://peanutbase.org/

2 https://www.arabidopsis.org/

3 https://sanger.ac.uk/

4 http://circos.ca/

5 https://www.r-project.org/

6 http://www.sigmaaldrich.com/

7 http://www.invitrogen.com/

8 http://www.idtdna.com/scitools/Applications/RealTimePCR

9 http://www.bio-rad.com
