# Supplementary material for: Genome-Wide Identification of Peanut KCS Genes Reveals That AhKCS1 and AhKCS28 Are Involved in Regulating VLCFA Contents in Seeds
Source: Front Plant Sci. 2020 May 7;11:406. doi: 10.3389/fpls.2020.00406 (PMC7221192; doi:10.3389/fpls.2020.00406)
Supplement: Supplementary file 1 [file Data_Sheet_1.pdf]

Supplementary Material

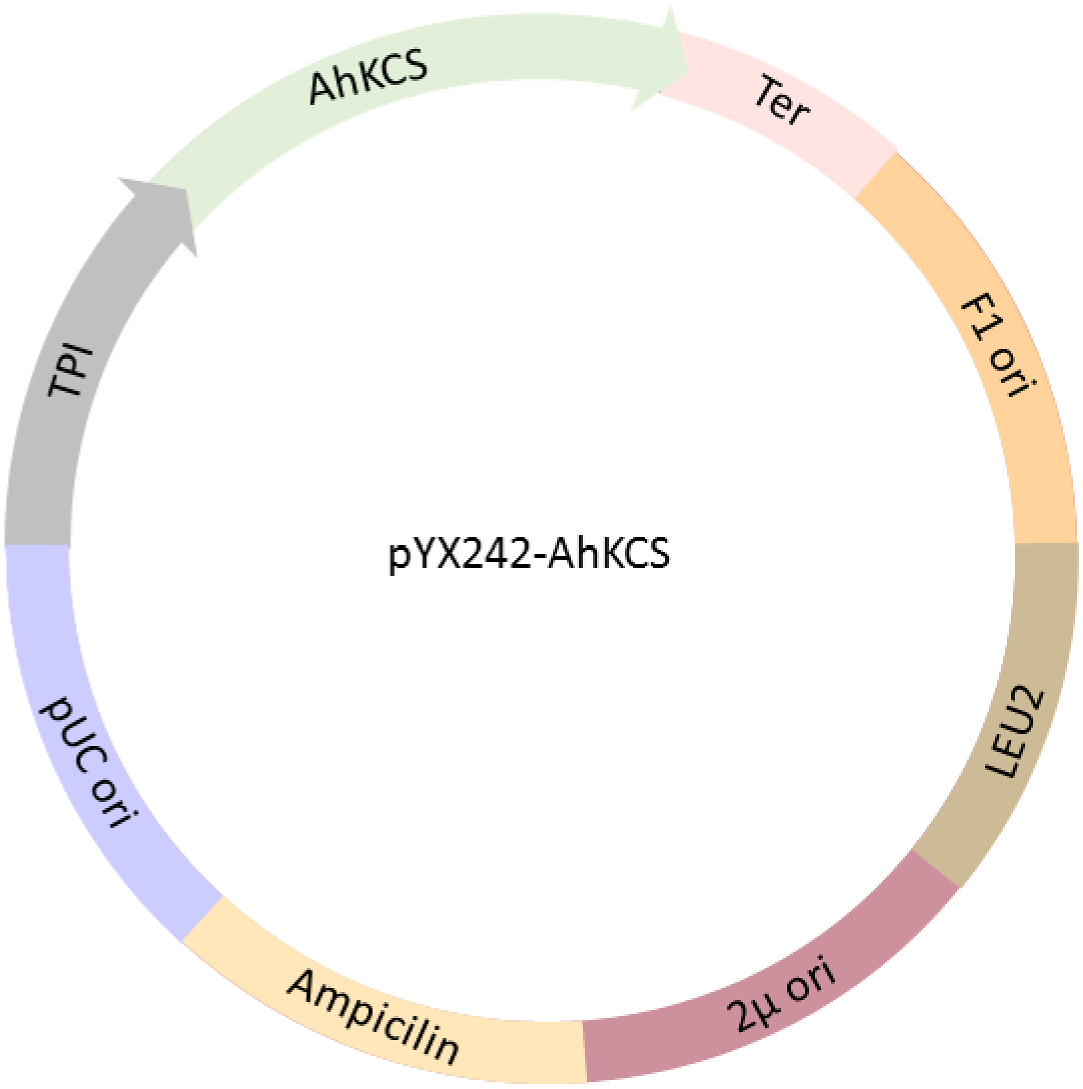

Figure S1. Constructs of pYX242-AhKCS genes

AhKCS1 and *AhKCS28* were inserted into pYX242 vector, respectively; TPI: triose phosphate isomerase promoter; Ter: terminator; f1 ori: origin of replication in phage; LEU2: LEU2 gene; 2μ ori: origin of replication in yeast; Ampicillin: anti-ampicillin gene; pUC ori: origin of replication in *E.coli*.

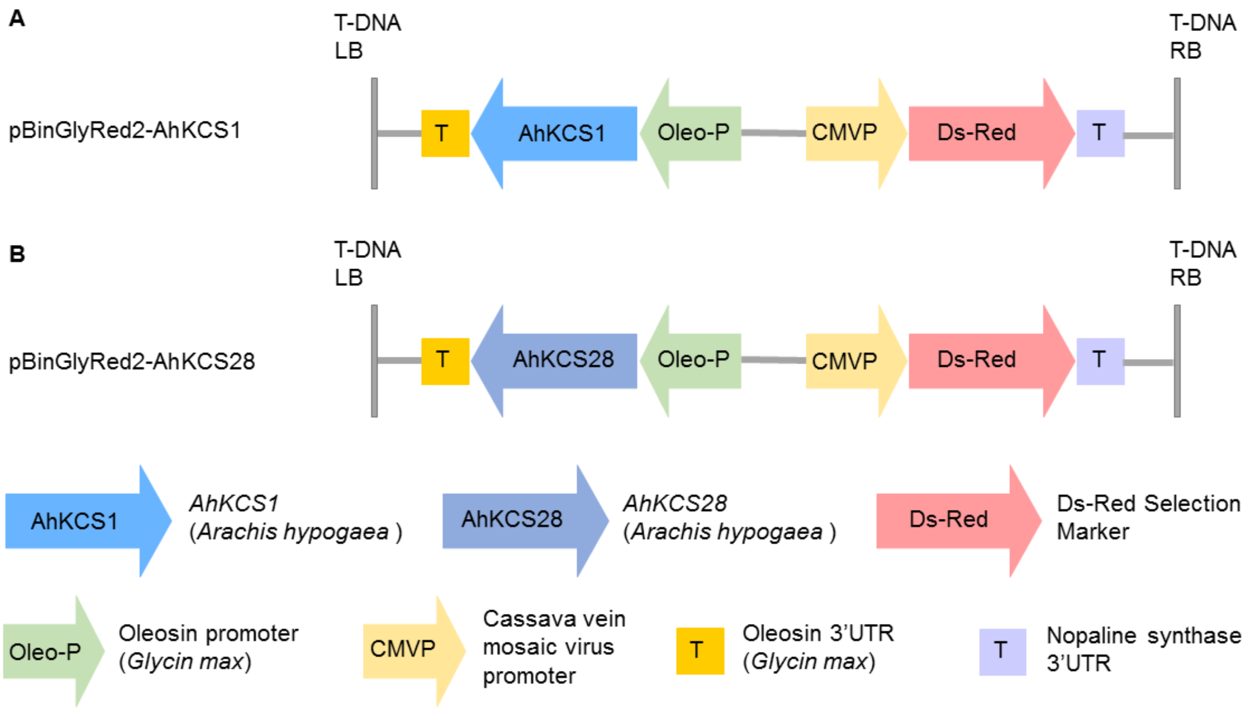

Figure S2. Constructs of pBinGlyRed2-AhKCS1 and pBinGlyRed2-AhKCS28

A. pBinGlyRed2-AhKCS1 contains seed-specific cassettes for the expression of *AhKCS1* gene and Ds-Red selection marker; B. pBinGlyRed2-AhKCS28 contains seed-specific cassettes for the expression of *AhKCS28* gene and Ds-Red selection marker. Selection was accomplished with a DsRed florescence marker. Constitutive and seed-specific promoters, 3 ’ UTR sequences and arrangements of cassettes are also shown.

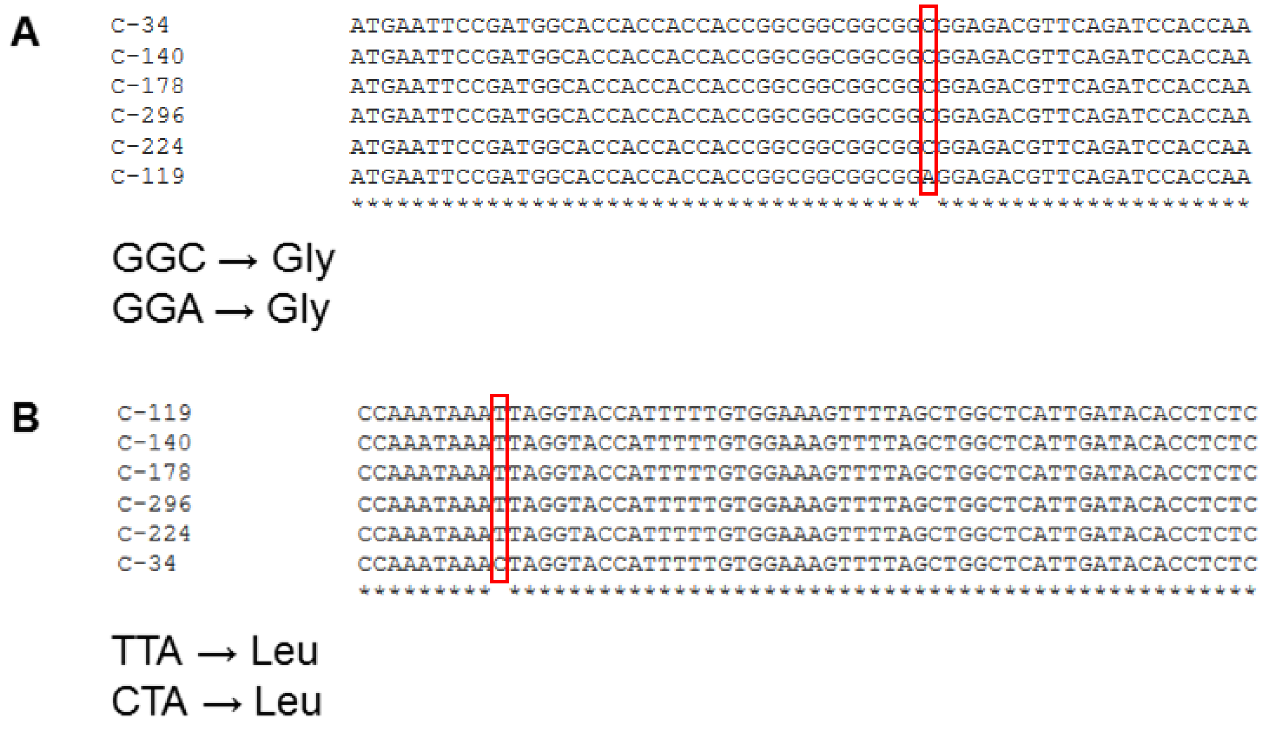

Figure S3. Alignment of sequences of *AhKCS17* (A) and *AhKCS23* (B) from peanut lines with different VLCFA contents.

Red boxes indicate mismatched bases.

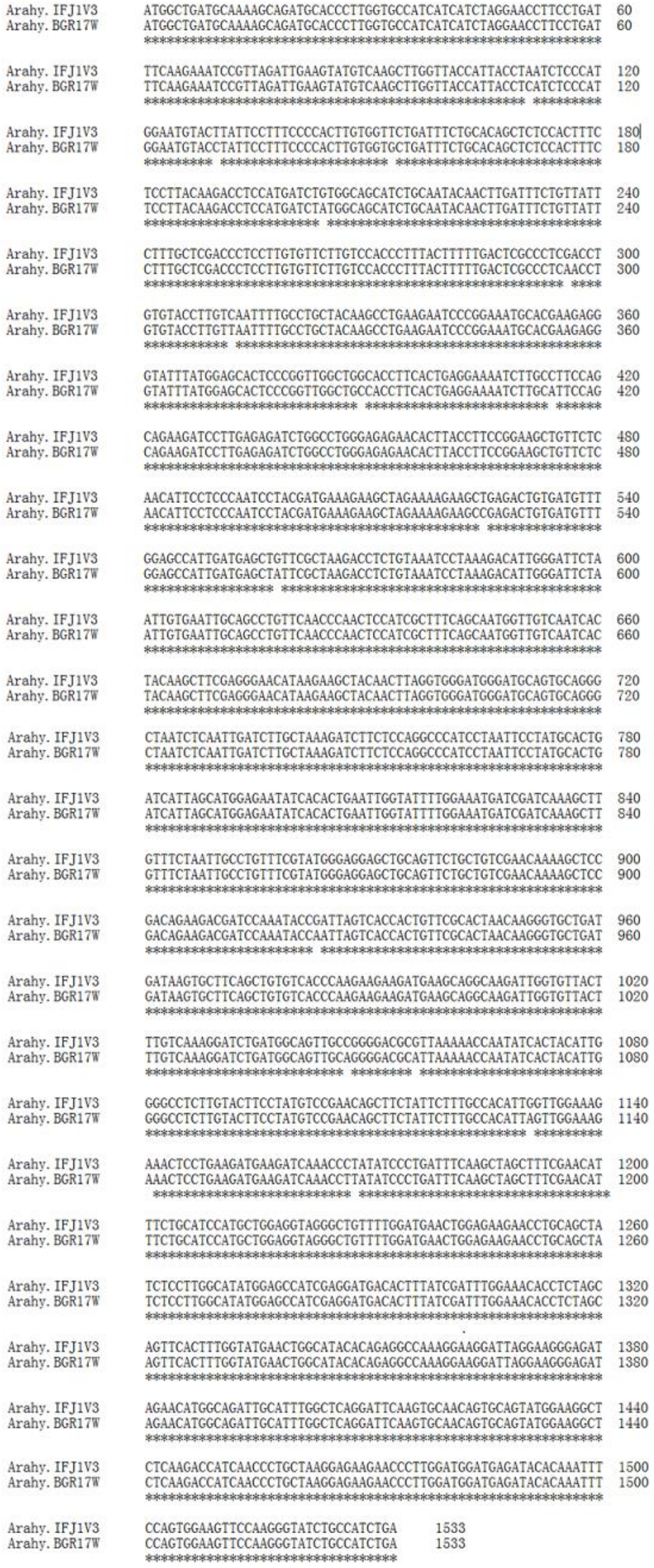

Figure S4. Alignment of sequences of *AhKCS1* and *AhKCS28* from peanut plants.

Arahy.IFJ1V3 is the *AhKCS1* gene; *Arahy.BGR17W* is the *AhKCS28* gene.
