# Supplementary material for: Genome-Wide Identification of Peanut KCS Genes Reveals That AhKCS1 and AhKCS28 Are Involved in Regulating VLCFA Contents in Seeds
Source: Front Plant Sci. 2020 May 7;11:406. doi: 10.3389/fpls.2020.00406 (PMC7221192; doi:10.3389/fpls.2020.00406)
Supplement: Supplementary file 2 [file Data_Sheet_2.pdf]

# *Supplementary Material*

**Table S1. Primers for qRT-PCR of *AhKCS* genes**

| Primer | Gene | Forward sequence (5'-3') | Reverse sequence (5'-3') |
|---|---|---|---|
| AhACTIN-RT | *AhACTIN* | TAAGAACAATGTTGCCATACAGA | GTTGCCTTGGATTATGAGC |
| AhKCS1/28-RT | *AhKCS1/AhKCS28* | AGGTAGGGCTGTTTTGGATG | TTCCTTTGGCCTCTGTGTATG |
| AhKCS4/17-RT | *AhKCS4/AhKCS17* | GGCGGCGGCGGAGACGTTCAG | ATGATTGACATGAGAGGAATC |
| AhKCS10/25-RT | *AhKCS10/AhKCS25* | ACAACCAAAAAAGCGACGATT | TTATTGCTGTTATTGTACTTTGCT |
| AhKCS13/29-RT | *AhKCS13/AhKCS29* | TTATGACCTTGCTACCGTCTTG | TTGAGGTGATCTTGAGGTTGG |
| AhKCS23-RT | *AhKCS23* | GGGCATAGCAGCAAATTCATC | CCCTATCACCTTTCTTAAGCCTC |

**Table S2. Primers for construction of yeast vectors**

| Primer | Gene | Forward sequence (5'-3') | Reverse sequence (5'-3') |
|---|---|---|---|
| AhKCS1/28-Y | *AhKCS1/AhKCS28* | CG<u>GAATTC</u>ATGGCTGATGCAAAAGCA (EcoRI) | CG<u>GGATCC</u>TCAGATGGCAGATACCC[illegible] |
| AhKCS4/17-Y | *AhyKCS4/AhKCS17* | TCC<u>CCCGGG</u>ATGAATTCCGATGGCACC (SmaI) | TCC<u>CCCGGG</u>CTATGTAACAACCTGC[illegible] |
| AhKCS10/25-Y | *AhyKCS10/AhKCS25* | CG<u>GAATTC</u>ATGGCAGATCCAAAGAAC (EcoRI) | CG<u>GGATCC</u>TCAATTTGAATTTATTG[illegible] |
| AhKCS13/29-Y | *AhyKCS13/AhKCS29* | CG<u>GAATTC</u>ATGGCGAATAACGAACGAGAT (EcoRI) | TCC<u>CCCGGG</u>TTAAGTGAGAGAGCG[illegible] |
| AhKCS23 | *AhyKCS23* | CG<u>GAATTC</u>ATGGAGATTCTGTTCTTATCTCTCT (EcoRI) | CG<u>GGATCC</u>TTACCCTTGTTCATCTT[illegible] |

**Table S3. Primers for cloning of *AhKCS* genes in *Arachis hypogaea***

| Primer | Accession | Forward sequence (5'-3') | Reverse sequence (5'-3') |
|---|---|---|---|
| AhKCS1-C | *Arahy.IFJ1V3* | ATTCCTCTTTTTCTCACTTCCTCTC | CCTCAGATGTCAGAAGTCACAGTAA |
| AhKCS4-C | *Arahy.T9PLK1* | TTGTGTCCCTCCCTCACTCCCTCC | GAACAGCTGAGACATTGTCTATTA |
| AhKCS10-C | *Arahy.1FMC3R* | ATTCGAAAACCCTAACCAACAACT | TTTAGTCACCCTCCCCCAAAA |
| AhKCS13-C | *Arahy.WQ1I1V* | ATCCATCACACACTCATATTAAA | AACTATTAACTTCAAAGAAAGACAA |
| AhKCS17-C | *Arahy.TIY3DH* | CACTCCCATTCAACCTTCTCCTCT | GAACAGCTGAGACATTGTCTATTA |
| AhKCS23-C | *Arahy.3ATP19* | TAATAATCTTTCTCTCCCTTAAT | TATTTCTTCGAGTTATTACATTTTC |
| AhKCS25-C | *Arahy.XI5WK7* | TTCCCCGTTTCACAATTCGAAAAC | TTTAGTCACCCTCCCAAAAAA |
| AhKCS28-C | *Arahy.BGR17W* | GCTGAGATTTCCTGGGTTTCATGTA | AGAACTCCTAAATTTGAACATGACT |
| AhKCS29-C | *Arahy.YW30D2* | GATCTTGATCTGGTGGCATGG | CTGCACGTGAGTTTTTACCTGGATA |

**Table S4. Information of *AtKCS* and *AtELO* genes from *Arabidopsis***

| ID | Accession |
|---|---|
| AtKCS1 | AT1G01120 |
| AtKCS2 | AT1G04220 |
| AtKCS3 | AT1G07720 |
| AtKCS4 | AT1G19440 |
| AtKCS5 | AT1G25450 |
| AtKCS6 | AT1G68530 |
| AtKCS7 | AT1G71160 |
| AtKCS8 | AT2G15090 |
| AtKCS9 | AT2G16280 |
| AtKCS10 | AT2G26250 |
| AtKCS11 | AT2G26640 |
| AtKCS12 | AT2G28630 |
| AtKCS13 | AT2G46720 |
| AtKCS14 | AT3G10280 |
| AtKCS15 | AT3G52160 |
| AtKCS16 | AT4G34250 |
| AtKCS17 | AT4G34510 |
| AtKCS18 | AT4G34520 |
| AtKCS19 | AT5G04530 |
| AtKCS20 | AT5G43760 |
| AtKCS21 | AT5G49070 |
| AtELO1 | AT1G75000 |
| AtELO2 | AT3G06460 |
| AtELO3 | AT3G06470 |
| AtELO4 | AT4G36830 |

**Table S5. Homologies (%) of *AhKCS* genes identified in peanut plants to *AtELO* genes from *Arabidopsis***

| | AtELO1 | AtELO2 | AtELO3 | AtELO4 |
|---|---|---|---|---|
| AhKCS1 | 14.60 | 15.10 | 16.89 | 15.02 |
| AhKCS2 | 15.52 | 16.60 | 15.62 | 15.38 |
| AhKCS3 | 11.93 | 17.71 | 17.44 | 16.57 |
| AhKCS4 | 16.67 | 16.45 | 16.35 | 16.97 |
| AhKCS5 | 11.45 | 14.06 | 14.20 | 13.45 |
| AhKCS6 | 16.44 | 14.52 | 14.22 | 15.79 |
| AhKCS7 | 13.33 | 13.26 | 13.58 | 15.79 |
| AhKCS8 | 14.20 | 13.02 | 12.79 | 14.36 |
| AhKCS9 | 17.70 | 16.18 | 17.19 | 17.54 |
| AhKCS10 | 15.91 | 14.56 | 16.30 | 15.33 |
| AhKCS11 | 11.66 | 15.91 | 16.67 | 13.33 |
| AhKCS12 | 16.67 | 15.58 | 16.35 | 13.76 |
| AhKCS13 | 14.10 | 14.05 | 15.07 | 13.54 |
| AhKCS14 | 15.27 | 13.49 | 13.85 | 16.10 |
| AhKCS15 | 15.95 | 16.60 | 15.62 | 14.96 |
| AhKCS16 | 11.48 | 18.59 | 17.88 | 15.96 |
| AhKCS17 | 16.67 | 16.45 | 16.35 | 16.97 |
| AhKCS18 | 13.02 | 16.59 | 14.83 | 12.44 |
| AhKCS19 | 16.00 | 19.07 | 19.88 | 18.33 |
| AhKCS20 | 16.44 | 14.52 | 14.22 | 15.79 |
| AhKCS21 | 11.93 | 16.67 | 13.95 | 15.47 |
| AhKCS22 | 15.57 | 15.85 | 15.95 | 17.44 |
| AhKCS23 | 13.44 | 14.85 | 14.84 | 14.66 |
| AhKCS24 | 14.77 | 14.06 | 13.95 | 14.92 |
| AhKCS25 | 11.94 | 15.49 | 15.54 | 15.27 |
| AhKCS26 | 17.70 | 16.18 | 17.19 | 17.54 |
| AhKCS27 | 15.15 | 15.19 | 17.04 | 15.33 |

| | | | | |
|---|---|---|---|---|
| AhKCS28 | 12.15 | 16.58 | 17.34 | 14.75 |
| AhKCS29 | 16.22 | 14.77 | 15.42 | 13.39 |
| AhKCS30 | 14.68 | 15.88 | 17.37 | 15.91 |
| AtELO1 | 100.00 | 28.57 | 27.61 | 60.29 |
| AtELO2 | 28.57 | 100.00 | 77.82 | 28.26 |
| AtELO3 | 27.61 | 77.82 | 100.00 | 28.10 |
| AtELO4 | 60.29 | 28.26 | 28.10 | 100.00 |

**Note:** Homology analysis was performed with ClustalW.

**Table S6. Correlation analysis between expression level of *AhKCS* genes and contents of VLCFAs in developing seeds.**

| Expression level of *AhKCS* | C20:0 | C20:1 | C22:0 | C24:0 | VLCFA |
|---|---|---|---|---|---|
| *AhKCS1/AhKCS28* | 0.62 | 0.65 | 0.90* | 0.71 | 0.78 |
| *AhKCS13/AhKCS29* | -0.1 | -0.02 | 0.37 | 0.03 | 0.13 |
| *AhKCS10/AhKCS25* | -0.17 | -0.09 | 0.3 | -0.03 | 0.06 |
| *AhKCS4/AhKCS17* | 0.34 | 0.38 | -0.11 | 0.27 | 0.15 |
| *AhKCS23* | 0.38 | 0.45 | 0.72 | 0.51 | 0.57 |

*Correlation is significant at the $p<0.05$ level

**Table S7. VLCFA contents (%) in peanut lines**

| Line | C20:0 | C20:1 | C22:0 | C24:0 | VLCFA |
|---|---|---|---|---|---|
| Zhonghua16 | 1.7±0.1 | 1.0±0.1 | 2.1±0.2 | 0.9±0.1 | 5.7±0.4 |
| C-34 | 1.4±0.1 | 0.6±0.1 | 1.9±0.3 | 0.7±0.1 | 4.6±0.3 |
| C-119 | 1.2±0.1 | 0.7±0.1 | 1.7±0.1 | 0.7±0.2 | 4.3±0.4 |
| C-140 | 1.2±0.1 | 0.8±0.1 | 2.0±0.5 | 0.7±0.2 | 4.7±0.8 |
| C-178 | 1.8±0.2 | 1.5±0.2 | 3.9±0.4 | 2.1±0.2 | 9.4±0.5 |
| C-296 | 2.0±0.2 | 1.4±0.1 | 4.2±0.6 | 2.1±0.2 | 9.6±0.7 |
| C-224 | 2.2±0.4 | 1.1±0.1 | 5.0±0.5 | 1.5±0.2 | 9.8±1.0 |

Means ± SD were calculated based on three independent replicates.

**Table S8. Correlation analysis between expression level of *AhKCS* and VLCFA content in different peanut lines.**

| Expression level of *AhKCS* | VLCFA |
|---|---|
| *AhKCS1/AhKCS28* | 0.93* |
| *AhKCS13/AhKCS29* | 0.22 |
| *AhKCS10/AhKCS25* | -0.42 |
| *AhKCS4/AhKCS17* | -0.44 |
| *AhKCS23* | -0.32 |

*Correlation is significant at the $p<0.05$ level

**Table S9. VLCFA contents (%) in mature seeds harvested from *Arabidopsis fae1/fad2* double mutant and $T_2$ transgenic lines.**

| Line | C20:0 | C20:1 | C22:0 | C22:1 | VLCFA | VLCSFA | VLCUFA | VLCS[illegible] /VLCU[illegible] |
|---|---|---|---|---|---|---|---|---|
| *fae1/fad2*-1 | 3.3 | 1.7 | ND | ND | 4.9 | 3.3 | 1.7 | |
| *fae1/fad2*-2 | 3.5 | 2.8 | ND | ND | 6.3 | 3.5 | 2.8 | |
| *fae1/fad2*-3 | 2.9 | 2.0 | ND | ND | 5.0 | 2.9 | 2.0 | |
| | | | | | | | | |
| BnA-1 | 0.6 | 9.4 | 1.1 | 16.1 | 27.2 | 1.7 | 25.5 | |
| BnA-2 | 0.5 | 13.4 | 1.6 | 22.1 | 37.5 | 2.1 | 35.5 | |
| BnA-3 | 0.6 | 10.2 | 1.4 | 20.4 | 32.6 | 2.0 | 30.6 | |
| BnA-4 | 0.5 | 10.2 | 0.8 | 14.0 | 25.5 | 1.3 | 24.2 | |
| BnA-5 | 0.6 | 12.0 | 1.2 | 14.8 | 28.6 | 1.8 | 26.8 | |
| BnA-6 | 0.6 | 9.7 | 1.3 | 17.7 | 29.3 | 1.9 | 27.4 | |
| BnA-7 | 0.4 | 12.8 | 1.8 | 13.9 | 28.8 | 2.1 | 26.7 | |
| BnA-8 | 0.1 | 7.5 | 0.3 | 8.3 | 16.2 | 0.4 | 15.8 | |
| BnA-9 | 1.1 | 11.9 | 1.2 | 15.0 | 29.2 | 2.3 | 26.9 | |
| BnA-10 | 0.7 | 12.4 | 1.6 | 14.2 | 28.9 | 2.3 | 26.6 | |
| | | | | | | | | |
| BnC-1 | 0.8 | 13.1 | 1.2 | 14.1 | 29.2 | 2.0 | 27.2 | |
| BnC-2 | 0.1 | 7.7 | 1.4 | 20.5 | 29.7 | 1.5 | 28.2 | |
| BnC-3 | 0.9 | 12.7 | 1.7 | 28.0 | 43.3 | 2.6 | 40.8 | |
| BnC-4 | 1.3 | 12.1 | 1.5 | 29.1 | 44.1 | 2.8 | 41.2 | |
| BnC-5 | 0.8 | 14.1 | 1.1 | 11.9 | 28.0 | 1.9 | 26.0 | |
| BnC-6 | 0.1 | 3.7 | 0.7 | 10.1 | 14.6 | 0.8 | 13.8 | |
| | | | | | | | | |
| Ah1-1 | 7.5 | 8.1 | 10.9 | ND | 26.5 | 18.4 | 8.1 | |
| Ah1-2 | 7.8 | 5.7 | 12.9 | ND | 26.4 | 20.7 | 5.7 | |
| Ah1-3 | 5.5 | 7.6 | 11.5 | ND | 24.6 | 17.0 | 7.6 | |
| Ah1-4 | 6.5 | 5.3 | 9.9 | ND | 21.8 | 16.5 | 5.3 | |
| Ah1-5 | 7.5 | 8.1 | 10.9 | ND | 26.5 | 18.4 | 8.1 | |

| | | | | | | | |
|---|---|---|---|---|---|---|---|
| Ah1-6 | 8.0 | 6.0 | 12.7 | ND | 26.7 | 20.7 | 6.0 |
| Ah1-7 | 7.4 | 8.8 | 9.1 | ND | 25.3 | 16.5 | 8.8 |
| Ah1-8 | 5.2 | 6.7 | 10.8 | ND | 22.7 | 16.0 | 6.7 |
| | | | | | | | |
| Ah28-1 | 8.2 | 6.7 | 11.7 | ND | 27.9 | 19.9 | 6.7 |
| Ah28-2 | 6.2 | 6.7 | 13.8 | ND | 28.0 | 20.0 | 6.7 |
| Ah28-3 | 7.2 | 8.1 | 11.7 | ND | 27.0 | 18.9 | 8.1 |
| Ah28-4 | 7.8 | 7.7 | 12.9 | ND | 30.6 | 20.7 | 7.7 |
| Ah28-5 | 6.8 | 7.3 | 10.8 | ND | 27.0 | 17.6 | 7.3 |

ND, not detected.

BnA: $T_2$ lines transformed with *BnaA.FAE1*; BnC: $T_2$ lines transformed with *BnaC.FAE1*; Ah1: $T_2$ lines transformed with *AhKCS1*; Ah28: $T_2$ lines trans
*AhKCS28*.

C20:0: arachidic acid; C20:1: eicosenoic acid; C22:0: behenic acid; C22:1: erucic acid; C24:0: lignoceric acid; C26:0: hexacosanoic acid.

VLCFA = C20:0 + C20:1 + C22:0 + C22:1 + C24:0 + C26:0; VLCSFA = C20:0 + C22:0 + C24:0 + C26:0; VLCUFA = C20:1 + C22:1.
